# Venom Gland Transcriptomic and Proteomic Analyses of the Enigmatic Scorpion *Superstitionia donensis* (Scorpiones: Superstitioniidae), with Insights on the Evolution of Its Venom Components

**DOI:** 10.3390/toxins8120367

**Published:** 2016-12-09

**Authors:** Carlos E. Santibáñez-López, Jimena I. Cid-Uribe, Cesar V. F. Batista, Ernesto Ortiz, Lourival D. Possani

**Affiliations:** 1 Departamento de Medicina Molecular y Bioprocesos, Instituto de Biotecnología, Universidad Nacional Autónoma de México, Avenida Universidad 2001, Apartado Postal 510-3, Cuernavaca, Morelos 62210, Mexico; santibanezlo@wisc.edu (C.E.S.-L.); jcidu@ibt.unam.mx (J.I.C.-U.); 2 Laboratorio Universitario de Proteómica, Instituto de Biotecnología, Universidad Nacional Autónoma de México, Avenida Universidad 2001, Apartado Postal 510-3, Cuernavaca, Morelos 62210, Mexico; fbatista@ibt.unam.mx

**Keywords:** enzymes, motifs, phylogenetic analysis, toxins, transcriptome

## Abstract

Venom gland transcriptomic and proteomic analyses have improved our knowledge on the diversity of the heterogeneous components present in scorpion venoms. However, most of these studies have focused on species from the family Buthidae. To gain insights into the molecular diversity of the venom components of scorpions belonging to the family Superstitioniidae, one of the neglected scorpion families, we performed a transcriptomic and proteomic analyses for the species *Superstitionia donensis*. The total mRNA extracted from the venom glands of two specimens was subjected to massive sequencing by the Illumina protocol, and a total of 219,073 transcripts were generated. We annotated 135 transcripts putatively coding for peptides with identity to known venom components available from different protein databases. Fresh venom collected by electrostimulation was analyzed by LC-MS/MS allowing the identification of 26 distinct components with sequences matching counterparts from the transcriptomic analysis. In addition, the phylogenetic affinities of the found putative calcins, scorpines, La1-like peptides and potassium channel κ toxins were analyzed. The first three components are often reported as ubiquitous in the venom of different families of scorpions. Our results suggest that, at least calcins and scorpines, could be used as molecular markers in phylogenetic studies of scorpion venoms.

## 1. Introduction

Despite the large number of studies available in the scorpion venom literature, concerning venom components and identification of their activities, only twelve scorpion families of the twenty recognized extant families [1,2] are currently studied (Table 1). While most of the studied scorpions belong to the family Buthidae, an increasing number of species from other families (i.e., Bothriuridae, Caraboctonidae, Hormuridae, Scorpionidae, Scorpiopidae, Urodacidae and Vaejovidae) are drawing the attention of researchers. In recent years, transcriptomic analyses of the venom gland of several scorpion species have been published, increasing our knowledge on the biodiversity of venom peptides, and allowing us to focus on the evolution of the genes coding for them (e.g., [1,3,4,5,6]). More recently, RNA-Seq has become the technology of choice in the study of venom gland transcriptomes, because it is a low-cost sequencing technology capable of producing millions of sequences at once [6], including those of the transcripts coding for several putative toxins or venom components that may not be easily detected in the venom for reasons including low expression levels, fast turnover, etc. (e.g., the difference between the number of scorpines found in the transcriptome and proteome of *Urodacus yaschenkoi* [6,7]).

Among the eight neglected scorpion families, Superstitioniidae Stahnke, 1940, stands out. This family, along with family Akravidae Levi, 2007 (a possible extinct family), are the only two monotypic scorpion families, meaning that each contains only one genus and one species (see [1]). The phylogenetic position of the family Superstitioniidae within the Tree of Life of scorpions suggests that it is closely related to the family Typhlochactidae Mitchell, 1971, a family of troglobitic scorpions endemic to Mexico [1], with both families included within the superfamily Chactoidea [8]; therefore, distantly related to buthid scorpions. The Superstitioniidae is geographically isolated in Arizona and the Baja Peninsula from its closest Typhlochactidae relatives in Eastern Mexico. The taxonomy and systematics of the family Superstitioniidae remains undisputed (see [8]). *Superstitionia donensis* Stahnke, 1940, the Superstition Mountains Scorpion, is a small (reaching a length of 30 mm in adults), shiny and spotted scorpion that inhabits arid deserts with sparse plant cover [9].

Given its uniqueness and phylogenetic position within the order Scorpiones (i.e., distantly related to buthids and closely related to troglobite scorpions, plus highly endemic), the scorpion species *S. donensis* is a perfect candidate to study its venom. In the present contribution, we analyze the venom gland transcriptome of *S. donensis*. We report 135 annotated venom transcripts, among which we found sequences that putatively code for toxins, plus other peptides and venom-specific proteins. We also identified 26 components for which the sequences determined through mass spectrometry analysis correspond to translated sequences found in the transcriptome. This work enriches our knowledge on venom peptides from unexplored scorpion families, and allows us to fill more pieces in the jigsaw puzzle of the scorpion venom evolution.

### Calcins, Scorpines, La1-Like and Potassium Channel κ Toxins in Scorpion Venoms

While most of the families of sodium and potassium channel toxins are the best-studied components of scorpion venoms, other constituents have recently received major attention. Among them are calcins, scorpines, La1-like peptides and the potassium channel κ toxins, which have been recently described for several species and have been associated with a number of envenomation effects produced by scorpion stings.

Calcins, toxins affecting calcium channels, are structurally characterized by an inhibitor cystine knot (ICK) motif, making them different from the sodium, chloride and potassium channel toxins [10,11,12]. They were first discovered in the venom of the buthid scorpions: *Hottentotta hottentotta* (=*Buthotus hottentotta*) in 1991 [13]; *Hottentotta judaicus* (=*Buthotus judaicus*) in 1996 [14]; and *Mesobuthus martensii* in 1997 [15]. The first reported peptide with affinity to ryanodine receptors (RyR) from non-buthid scorpions was found in the venom of *Pandinus imperator* in 1992 [16]. Since then, calcins have been found in the venom of several species belonging to nine families, out of the 11 thus far studied (see Table 2). Ma et al. [3] analyzed the phylogenetic affinities of calcins (those available at that time) and their results showed the differences between buthid and non-buthid calcins, and that maurocalcin had an independent origin from the calcins of the rest of the Scorpionidae species.

Scorpines are peptides with dual activity: they are cytolytic (or antimicrobial), but also contain a potassium channel-blocking domain [17]. The first scorpine discovered was isolated from the venom of *Pandinus imperator* [18]. Subsequently, a wide phylogenetic distribution of these peptides in other scorpion families was established [19] (Table 2). Recently, this subfamily was shown to have an independent origin from the rest of the potassium channel toxins [5].

La1-like, long-chain peptides with eight cysteines [11], are also ubiquitous in scorpion venoms. They have been described in the venom of species belonging to 10 out of the 11 families studied so far (Table 2); and interestingly their function remains unknown. Ma et al. [3] also revised the phylogenetic status of these peptides, and found the presence of two main clades, plus four La1-like peptides from the venom of *Scorpiops jendeki* clustered independently from their phylogenetic origin, which would suggest multiple gene duplications. Later, Sunagar et al. [4] revised again these peptides, though not including the sequences obtained in the previous analysis (i.e., [3]). Their results showed that these peptides have multiple origins, and therefore do not mirror the scorpion phylogenetics (i.e., follows the proposed phylogenetic history of scorpions).

Finally, and unlike the other components mentioned before, the potassium channel κ toxins subfamily has not been found in many of the scorpion venoms studied thus far. These toxins, with a distinct CSαα motif [17] and low activity on potassium channels have been described in only a few species belonging to three scorpion families (Table 2).

In the present contribution, we revise the status of these components (calcins, scorpines, La1-like peptides and potassium channel κ toxins) using phylogenetic analyses under Bayesian inference. We propose motifs for those clades recovered with high posterior probabilities. This will contribute to establish a more complete and stable classification, and will help to categorize newly discovered components from different scorpion venoms in the future.

## 2. Results and Discussion

### 2.1. S. donensis Venom Gland Global Transcriptomic Analysis

**Figure 1:**
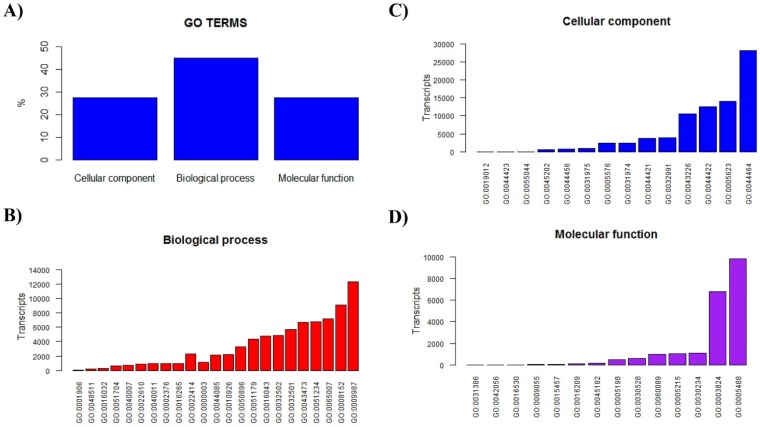
(**A**) Distribution of annotated sequences from the venom gland transcriptome of *S. donensis* according to Gene Ontology (GO) terms. The category designated by GO as “Biological process” was the most diverse. (**B**–**D**) Distribution of the most represented categories within each GO term (GO numbers shown).

After sequencing, assembly and cleaning, 16,145,663 reads were obtained corresponding to 219,073 transcripts. From them, a total of 45,979 where identified matching sequences in databases, with an N50 of 468 bp. A subgroup of 9930 was annotated, of which 1719 matched known arthropod sequences. Few transcripts (120) were classified as having identity to annotated genes or transcripts from arachnids, in particular, 62 were from scorpions; however, this seems to be the result of incompleteness of the databases against which the sequences are compared. Figure 1 shows the most abundant GO-term categories found in the transcriptome analysis of the venom gland of *S. donensis*.

### 2.2. The Repertoire of Venom-Specific Transcripts in S. donensis

**Figure 2:**
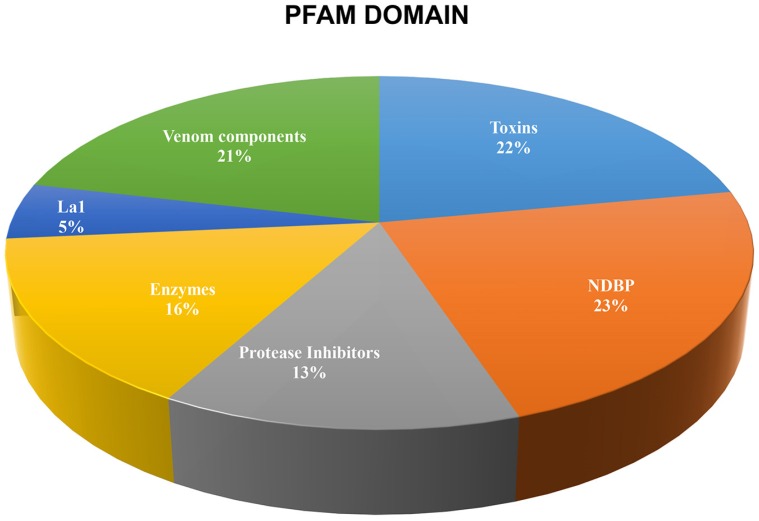
Relative proportion (expressed as percentages) of the Pfam domains of the 135 annotated transcripts, which putatively code for venom components found in the venom gland transcriptome analysis of *S. donensis*. The category Toxins includes putative Na^+^, K^+^ and Ca^2+^ toxin channels peptides; the category NDBPs (Non-Disulfide-Bridged Peptides) includes all possible NDBPs peptides even when no Pfam domain was found; the category Protease Inhibitors includes Ascaris-Type and Kunitz-Type inhibitors; the category La1 includes putative La1-type peptides; the category Enzymes includes all possible peptides with venom enzymatic activity; and the category Other Venom Components includes putative venom proteins and possible CAP peptides.

Following the scheme presented in recent venom gland transcriptome analyses [6,11], we report here 135 sequences putatively coding for the following known venom peptides and proteins (Figure 2):

#### 2.2.1. Toxins

Scorpion venoms are composed mainly by two distinct types of fractions: the toxic and cytolytic peptides and the nontoxic [20], which includes a complex mixture of different enzymes. In addition they might contain carbohydrates, lipids, free amines, nucleotides and other components with unknown function [5]. The toxic fraction of scorpion venom is historically the most studied one, due to the scorpion’s clinical relevance. These toxins affect sodium, potassium, calcium and chloride channels; therefore, they have been employed as tools to study the physiology, or the three-dimensional level of the molecular structure of these channels (e.g., [21,22]). In the transcriptome analysis of the venom gland of *S. donensis*, we found 30 transcripts putatively coding for ion channel-acting toxins, representing 22% of all transcripts (Figure 2). This is congruent with other transcriptomic analyses from non-Buhtidae species like the members of families Caraboctonidae [23]; Urodacidae [6]; and Vaejovidae [11].

##### Sodium Channel Toxins

**Figure 3:**
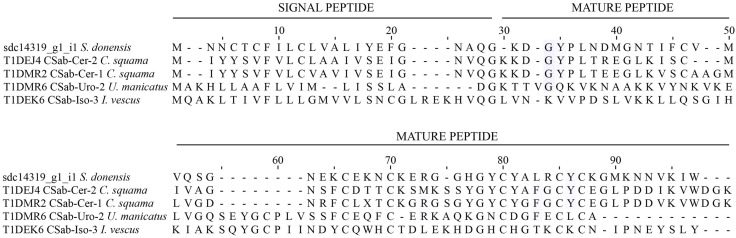
Sequence alignment of components with identity with sodium channel toxins (cysteine-stabilized α/β motif, CS αβ, indicated as CSab in the toxin names) found in the transcriptome analysis of the venom gland of *S. donensis* and those that were similar. Unitprot entry numbers precede the toxins’ names: (a) Component sdc14319_g1_i1, translated ORF; (b) CSab-Cer-2 from *Ce. squama*; (c) CSab-Cer-1 from *Ce. squama*; (d) CSab-Uro-2 from *Urodacus manicatus*; and (e) CSab-Iso-3 from *Isometroides vescus*.

The sodium channel toxins are modifiers of the gating mechanism of the channel [24], and are responsible for the neurotoxic symptoms during envenomation [5,25]. Therefore, these toxins (along with those affecting potassium channels) are by far the best studied in scorpion venoms. There are currently more than 520 sequences of toxins (or putative toxins) listed in the InterProt database [26]. Most of them (99%) belong to buthid scorpions (for recent reviews, see [5,17]). Several non-Buthidae transcriptomic analyses (e.g., [6,11,23]) have reported the presence of a low number of sodium channel toxin-coding transcripts. We, unlike other studies (e.g., [6,10]), found eight transcripts for this kind of toxin: (a) one putatively coding for a component labeled sdc10528_g1_i1 with 43% identity with the precursor of Toxin To9, deduced from a cDNA cloned from *Tityus obscurus* [27]; (b) component sdc14462_g1_i1 with 54% identity with the mature chain of Altitoxin, obtained from the venom of *Parabuthus transvaalicus* [28]; (c) component sdc14462_g1_i2 with 57% identity with the mature chain of Birtoxin, also from the venom of *P. transvaalicus* [29]; (d) component sdc15193_g1_i1 with 61% identity with the precursor of Toxin Cll7, deduced from a cDNA cloned from *Centruroides limpidus* (Uniprot accession number: P59865); (e) component sdc16570_g1_i1 with 44% identity with the precursor of Toxin TdNa8, deduced from a cDNA cloned from *Tityus discrepans* [30,31]; (f) component sdc21236_g1_i1 with 42% identity with the precursor of the Neurotoxin LmNaTx30, deduced from the transcriptome analysis of the venom gland of *Lychas mucronatus* [32]; (g) component sdc10528_g1_i1 with 43% identity with the precursor of the Toxin Pg8, isolated from the venom (but also deduced from the cloned cDNA) of *Parabuthus granulatus* [33]; and (h) sdc14319_g1_i1 with 48% identity with the precursor Csab-Cer-2 deduced from the transcriptomic analysis of the venom gland of *Cercophonius squama* [4]. In Figure 3 we show only some representative examples of these Na^+^-channel peptides. In addition to this, one sequence had hits with 50% identity with the precursor of the Lipolysis activating peptide 1 (LPV1) alpha chain, isolated (and deduced from a cloned cDNA) from the venom of *Buthus tunetanus* (=*Buthus occitanus tunetanus*) [34]. These peptides share sequence identity with the sodium channel toxins; however, unlike them, LPV1s lack a cysteine in their sequence resulting in a reduced number of disulfide bridges and distinct interchain bridges [34].

##### Potassium channel toxins

Peptides acting on K^+^ channels vary in size from 23 to up to 64 amino acids and are classified based on primary sequence and disulfide bond connectivity [5,35]. Along with the sodium channel toxins mentioned above, potassium channel toxins are also well studied. In the InterProt database, there are nearly 230 sequences coding for short and long potassium channel toxins [36,37]. Most of them (78%) belong to buthid scorpions. Four families have been recognized for these toxins: the α-, β- and γ- families, stabilized by the CSαβ motif, and the κ- family [5].

**Figure 4:**
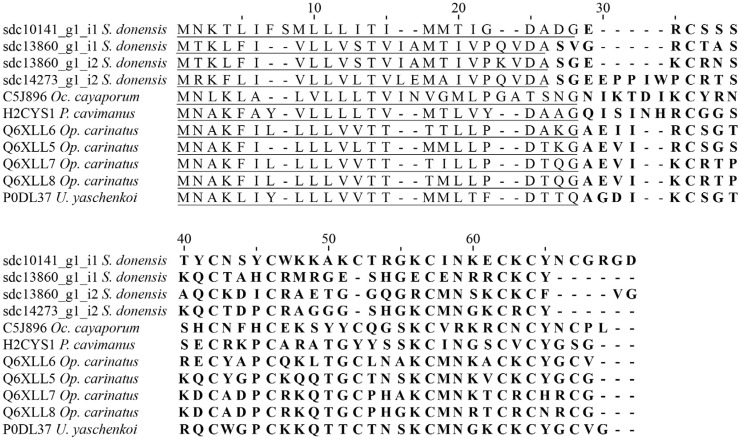
Amino acid sequences of the translated transcripts showing identity with the αKTx subfamily 6, found in the transcriptome analysis of the venom gland of *S. donensis*, aligned to similar sequences. Unitprot entry numbers precede the species’ names: C5J896 (potassium channel toxin αKTx 6.16); H2CYS1 (αKTx-like peptide); Q6XLL6 (potassium channel toxin αKTx 6.9); Q6XLL5 (Potassium channel toxin αKTx 6.10); Q6XLL7 (potassium channel toxin αKTx 6.8); Q6XLL8 (Potassium channel toxin αKTx 6.7); and P0DL37 (potassium channel toxin αKTx 6.21). The predicted signal peptide is underlined and the mature peptide is in bold.

Our results were consistent with previous scorpion venom gland transcriptomic analyses (e.g., that of *U. yaschenkoi* [6]). We found 11 sequences showing identity with seven different potassium channel toxins (members of α- and κ- families). Ten sequences coding for six putative α-KTxs were found: (a) components sdc13860_g1_i2 and sdc14273_g1_i2 had hits with 39% and 48% identity, respectively, with the precursor of the potassium channel toxin αKTx 6.7 deduced from a cDNA cloned from the venom of *Opistophthalmus carinatus* [38]; (b) components sdc13860_g1_i1 and sdc10141_g1_i1 had hits with 42% and 46% identity, respectively, with the precursor of the potassium channel toxin αKTx 6.10 also deduced from a cDNA cloned from the venom of *O. carinatus* [38] (Figure 4 shows the alignment of the sequences with identity with members of the αKTx subfamily 6); (c) components sdc26193_g1_i1 and sdc13949_g1_i1 had hits with 60% and 70% identity, respectively, with the precursor of the Toxin LmKTx 8 deduced from a cDNA cloned from *Lychas muronatus* [39]; (d) components sdc9772_g1_i2 and sdc9772_g1_i1 had hits with 40% and 42% identity, respectively, with the precursor of a potassium channel toxin deduced from a cDNA cloned from *U. yaschenkoi* [40]; (e) component sdc14273_g1_i1 had hits with 44% identity with the precursor of the potassium channel toxin αKTx 12.5 deduced from the transcriptome analysis of the venom gland of *L. mucronatus* [32]; and (f) component sdc13973_g1_i2 had hits with 46% identity with the precursor of a potassium channel toxin named Tbah02745 deduced from the transcriptome analysis of the venom gland of *Tityus bahiensis* [41].

Finally, one component (sdc14251_g2_i1) had hits with 58% identity with the precursor of the potassium channel κKTx 5.1 deduced from a cDNA cloned (and later isolated) from *Heterometrus laoticus* [42].

##### Scorpine-like peptides

**Figure 5:**
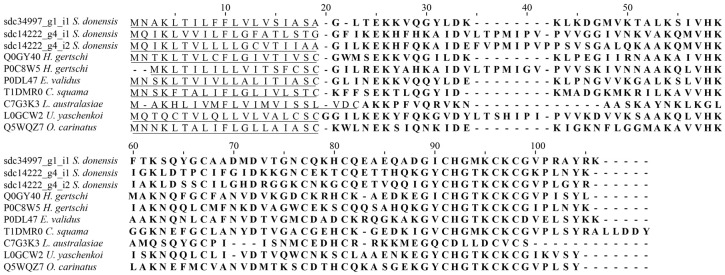
Sequence alignment of components with identity with Scorpines found in the transcriptome analysis of the venom gland of *S. donensis*. Peptide sequences were generated by translation from the reported transcripts. For comparative purposes, other known sequences are included (Unitprot entry numbers in brackets). Components sdc34997_g1_i1, sdc14222_g4_i1 and sdc14222_g4_i2; Hge scorpine and He scorpine-like 2 from *Ho. gertschi* (Q0GY40 and P0C8W5 respectively); Scorpine-like peptide Ev37 from *E. validus* (P0DL47); CSab-Cer-6 from *Ce. squama* (T1DMR0); β-KTx-like peptide LaIT2 from *Liocheles australasiae* (C7G3K3); Antimicrobial peptide scorpine-like 2 from *U. yaschenkoi* (L0GCW2); and Opiscorpine 3 from *Op. carinatus* (Q5WQZ7). The predicted signal peptide is underlined and the mature peptide is in bold.

These are 59–75-amino acid-long peptides stabilized by three disulfide bridges [19]. As mentioned before, they were originally classified as members of the β-KTx family (e.g., [19]), although they were also considered “orphan” peptides because their function was not completely identified at that time. These peptides possess two different structural and functional domains: one domain with antimicrobial and cytoltic activity and another with potassium channel-blocking activity [17]. However, recent phylogenetic analysis of scorpion toxins [5] and unpublished analysis considering only the potassium channel-blocking domain showed independent origin from the rest of the potassium channel toxin families; therefore, they should be considered as an independent subfamily. Seven sequences encoding putative scorpines were found in our analysis: (a) component sdc34997_g1_i1 with 59% identity with the precursor of the Hge scorpine deduced from the transcriptome analysis of the venom gland of *Hoffmannihadrurus gertschi* (=*Hadrurus gertschi*) [23]; (b) components sdc2871_g1_i1, sdc14222_g4_i1, sdc14222_g4_i2 and sdc20456_g1_i1 with 59%, 53%, 64% and 64% identity, respectively, with the precursor of the Hg scorpine-like 2 deduced also from the transcriptome *Ho. gertschi* [23]; (c) component sdc4553_g1_i1 with 58% identity with the precursor of the antimicrobial peptide scorpine-like 1 deduced from a cDNA cloned from *U. yaschenkoi* [7]; and (d) component sdc23468_g1_i1 with 60% identity with the precursor of the Csab Uro 4 deduced from the transcriptomic analysis of the venom gland of *U. manicatus* [4]. In Figure 5, a few representative sequences are shown and only the non-buthid scorpine-like peptides were included in this figure for comparison purposes. Our results were consistent with the number of scorpine-like peptides found in transcriptomic analyses published before [6,11].

##### Calcins

**Figure 6:**
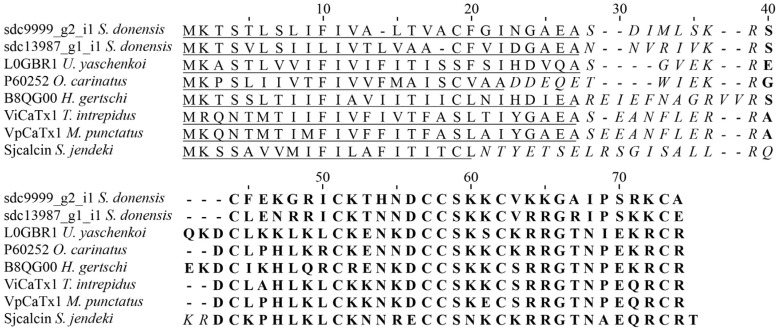
Sequence alignment of components with identity with calcins found in the transcriptome analysis of the venom gland of *S. donensis* and those that were similar. The transcripts were translated to generate the peptidic precursor sequences. Unitprot entry numbers in brackets. Components sdc9999_g2_i1 and sdc13987_g1_i1; Calcium channel toxin like 20 from *Urodacus yaschenkoi* (L0GBR1); Hadrucalcin from *Hoffmannihadrurus gertschi* (B8QG00); ViCaTx1 from *Thorellius intrepidus* [11]; β-KTx-like peptide LaIT2 from *Liocheles australasiae* (C7G3K3); Antimicrobial peptide scorpine-like 2 *Urodacus yaschenkoi* (L0GCW2); and Opiscorpine 3 from *Op. carinatus* (Q5WQZ7). The predicted signal peptide is underlined; the mature peptide is in bold and the propeptide is in italics.

As mentioned before, these peptides have been isolated from the venom of all scorpions, but they are more abundant in non-buthid scorpions. Their structure is different from the toxins affecting other ion channels (i.e., sodium, potassium and chloride) because they lack a cysteine-stabilized α/β motif [5]. Instead, they have an Inhibitor Cystine Knot (ICK) motif, like in the peptides found in spider and snail venoms affecting the calcium channel [6,43]. Calcins recognize ryanodine-sensitive calcium channel (RyRs) of the endoplasmic and sarcoplasmic reticula of skeletal and cardiac muscle [6,10,12,16]. In the transcriptome analysis of the venom of *S. donensis*, we found two sequences that putatively code for calcin peptides similar to those found in other scorpions: (a) component sdc9999_g2_i1 with 39% identity with the precursor of the Ca-like-20 deduced from a cDNA cloned from *Urodacus yaschenkoi* [7]; and (b) component sdc13987_g1_i1 with 45% identity with the precursor of Opicalcin 1 deduced from a cDNA cloned from the venom of *Op. carinatus* [44]. Figure 6 shows the sequence alignment of these two components and those that were similar. We found a sequence (sdc21328_g1_i1) with 62% identity to the precursor of the U8 agatoxin Ao1a (not a member of the Calcin family) deduced from a cloned cDNA from *Agelena orientalis* [45]. The data we report here indicate the presence of two different types of calcins: one with seven cysteines and the other with eight cysteines.

#### 2.2.2. Non-Disulfide-Bridged Peptides (NDBPs)

Although often scarce in toxins, the venom of the non-Buthidae scorpions is usually rich in another large group of peptides characterized by the lack of cysteines, called the Non-Disulfide-Bridged Peptides (NDBPs). They have been shown to display multiple biological activities, including antimicrobial, cytolytic, anti-inflammatory, among others [46], therefore attracting major attention as potential clinical and therapeutic candidates in drug development [47]. The NDBPs appear to be the most abundant components in the venom of the non-buthid scorpions [11]. In our analysis, we found 31 transcripts (23% of all transcripts reported here; Figure 2) with identities to those encoding for 19 previously reported NDBPs. Among those peptides, only one sequence (sdc4413_g1_i1) had hits with 58% identity with the precursor of the venom antimicrobial peptide 6 deduced from a cDNA cloned from *Mesobuthus eupeus* [48,49], an amphipathic peptide exhibiting extensive cytolytic activity found in a buthid scorpion.

**Figure 7:**
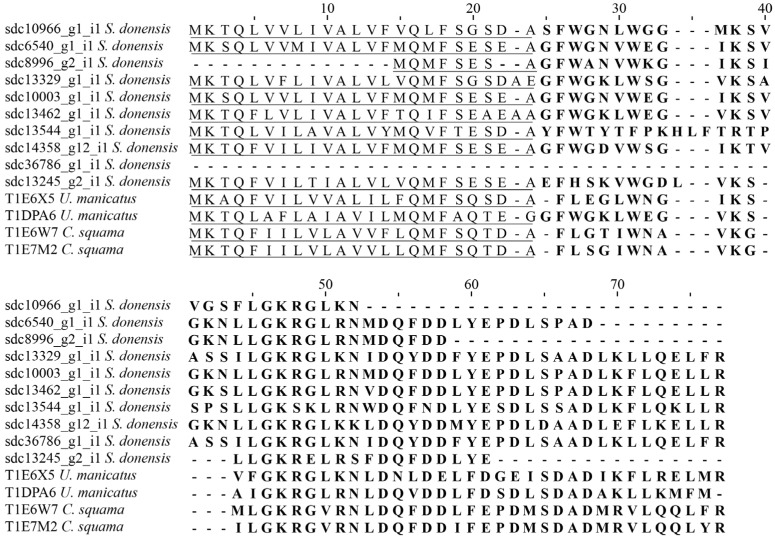
Sequence alignment of components with identity with Non-Disulfide-Bridged Peptides found in the transcriptome analysis of the venom gland of *S. donensis*. The sequences derived from transcripts were translated to show the precursor peptidic sequences. For comparative purposes other known sequences are included (Unitprot entry numbers in brackets): CYLIP-Uro-1 and CYLIP-Uro-3 from *U. manicatus* (T1E6X5 and T1DPA6, respectively); and CYLIP-Cer-2 and CYLIP-Cer-3 from *Ce. squama* (T1E6W7 and T1E7M2, respectively). The predicted signal peptide is underlined and the mature peptide is in bold.

Among the rest of the NDBPs found in this study (but see Supplementary Table S1 for the complete list), two components, sdc24668_g1_i1 and sdc36542_g1_i1, had hits with 60% and 68% identity, respectively, with the precursor of the amphipathic peptide CT1 deduced from a cDNA cloned from *Vaejovis mexicanus* [50]; and four transcripts had hits (with identities greater than 56%) with several precursors named amphipathic peptides CT2 from different scorpion species (all deduced from cDNA) such as those cloned from *Scorpiops tibetanus* [51]; *Mesomexovis subcristatus* (=*Vaejovis subcristatus*) [50], and *Vaejovis smithi* [50]. Four more components (see Supplementary Table S2) had hits with two different amphipathic peptides deduced from cDNA cloned from *Mesomexovis punctatus* (=*Vaejovis punctatus*) [52]; ten components had hits with identities between 42% and 60% with four different CYLIP peptides deduced from the transcriptome analyses of the venom glands of *U. manicatus* and *Ce. squama* [4] (Figure 7). Other components had hits with several NDBPs reported from the venom gland of several scorpion species including *O. madagascariensis*, *Ho. gertschi*, *Heterometrus petersii* and *V. mexicanus* (see Supplementary Table S1).

#### 2.2.3. La1-Like Peptides

La1-like peptides have been found more and more frequently in the scorpion venoms. The original La1 peptide was isolated from the venom of *Liocheles australasiae* as its most abundant component [53]. The family of scorpion venom peptides defined by La1 [54] consists of long chain (73–100 aa) peptides stabilized by four disulfide bridges [6,11]. The biological activity of these peptides or their role in the venom remains unknown. In our analysis, seven sequences encoding six putative mature La1-like peptides were found: (a) sdc12897_g1_i1 with 48% identity with the precursor of La1-like protein 13 deduced from the transcriptomic analysis of the venom gland of *Urodacus yaschenkoi* [6]; (b) sdc14036_g1_i1 with 56% identity with the precursor of La1-like protein 15 deduced from the transcriptome analysis of the venom gland of the same species; (c) sdc10164_g1_i1 and sdc13004_g1_i1 with 40%–41% identity with the precursor of a putative secreted protein deduced from cDNA cloned from the venom of *Opisthacanthus cayaporum* [55]; (d) sdc7328_g1_i1 with 53% identity with the precursor of the SV-SVC-Cer 1 deduced from the transcriptome analysis of *Ce. squama* [4]; (e) sdc14589_g1_i1 with 47% identity with the precursor of a Toxin like protein 14 deduced from cDNA and a transcriptomic analysis of the venom gland of *U. yaschenkoi* [6,7]; and (f) sdc5116_g1_i1 with 42% identity with the precursor of the venom protein 7 deduced from cDNA cloned from *Mesobuthus eupeus* (with Uniprot accession number E4VP44). These results were consistent with those reported in the transcriptome analyses of two species of *Scorpiops* [3,4,5,6,7,8,9,10,11,12,13,14,15,16,17,18,19,20,21,22,23,24,25,26,27,28,29,30,31,32,33,34,35,36,37,38,39,40,41,42,43,44,45,46,47,48,49,50,51,52,53,54,55,56], and of *U. yaschenkoi* [6].

#### 2.2.4. Enzymes

Enzymes have been reported to be abundant components of scorpion venoms [6]. Interestingly, and not in line with the findings in other transcriptome analyses (e.g., [6]), where sequences coding for enzymes represented about a third of the transcripts, our results show that only 16% (21 transcripts; Figure 2) putatively code for enzymes, including phospholipases, hyaluronidases and peptidases. Within these transcripts we report five transcripts with identities with one putative phospholipase deduced for one scorpion species, and six transcripts with identity with two putative hyaluronidases deduced from the venom of two scorpion species (but see Supplementary Table S1 for the complete list). Among the phospholipases, we found five transcripts with 42%–55% identity with the precursor of the Phospholipase A2 deduced from the transcriptome analysis of the venom gland of *Ho. gertschi* [23]. The six transcripts with identities with two putative hyaluronidases found were: (a) three transcripts with 42%–53% identity with the precursor of the Hyaluronidase 1 deduced from cDNA cloned from the venom of *Mesobuthus martensii* [57]; and (b) three transcripts with 46% identity (all) with the precursor of the Hyaluronidase 2 deduced from cDNA cloned from the venom of *Tityus serrulatus* [58].

#### 2.2.5. Protease Inhibitors

Two different types of protease inhibitors have been isolated from the venom of scorpions.

*Ascaris-type*: These serine protease inhibitors proposed to be modulators of protease activity, have been found in different organisms protecting against toxins and other components from unwanted degradation [59]. They have a common structure with short β strands stabilized by five disulfide bridges [59,60]. Unlike other venom gland transcriptomes reported [6,11] where less than ten sequences were reported, 18 sequences encoding seven different ascaris-type peptides were obtained from the transcriptome analysis of the venom gland of *S. donensis*. Among these peptides (see Supplementary Table S1 for the complete list), we found 16 transcripts with identities with three putative Ascaris-type peptides deduced from cDNA and transcriptome analyses of three scorpion species including: the precursor of a cysteine-rich venom protein and a putative salivary secreted serine protease inhibitor deduced from cDNA cloned from *Pandinoides cavimanus* (=*Pandinus cavimanus*) [61]; and the precursor of the venom peptide SjAPI and SjAPI 2 deduced from cDNA cloned from *Scorpiops jendeki* [59]. Two transcripts had hits with peptides found in the saliva of a mosquito (*Stegomyia albopicta*) and a mite (*Rhipicephalus pulchellus*), respectively.

*Kunitz- type*: These peptides are known for their protease inhibitor activity (as trypsin inhibitors), but they also block (very weak) the Kv1.3 potassium channels [62]. In the transcriptome analysis of the venom gland of *S. donensis* we found two sequences: (a) sdc12570_g1_i1 had hits with 71% identity with the precursor of Kunitz-type serine protease inhibitor Hg1 deduced from the transcriptome of *Ho. gertschi* [23,62]; and (b) sdc31500_g1_i1 with 47% identity with the precursor of the peptide HW11c39 deduced from the cDNA cloned from the tarantula *Haplopelma schmidti* [63].

#### 2.2.6. Other Venom Components

*CAP superfamily*: The proteins and peptides included in this superfamily have been more frequently found in the venom of scorpions. They are cysteine-rich secretory proteins, with extracellular endocrine or paracrine functions; or they might act as proteases or protease inhibitors [64]. Within this category, we also include allergens, peptides found in the venom of several arthropods (insects, arachnids and myriapods). We report 13 sequences encoding seven putative CAP peptides (see also Supplementary Table S1). Component sdc13900_g1_i1 had hits with 32% identity with the precursor of CAP-Iso-1 deduced from the transcriptome analysis of *Isometroides vescus* [4]. Three components had hits with 24% to 27% identity with the precursor of CAP-Lyc-1 deduced from the transcriptome analysis of the venom gland of *Lychas buchari* [4]. Two components had hits with 54% and 79% identity with the precursor CAP-Uro-1 deduced from the transcriptome analysis of *U. manicatus* [4]. Two components had hits with 32% identity (both) with the precursor Tbah00853 deduced from the transcriptome analysis of *T. bahiensis* [41]; and two components had hits with 59% identity (both) with the precursor of a putative cysteine-rich secretory peptide deduced from the transcriptome analysis of *Hottentotta judaicus* [65].

*Venom components*: Other transcripts potentially coding for venom proteins not covered in the above-described categories represent 21% of those annotated in the transcriptome analysis (Figure 2, but see also Supplementary Table S1). We found four sequences with identities ranging from 42% to 46% with the precursor of venom insulin-like growth factor binding protein 1 deduced from cDNA cloned from *Mesobuthus martensii* (only submitted to GenBank); three sequences with 44%–51% identity with the precursor of Tbah01400 deduced from the transcriptome analysis of the venom gland of *Tityus bahiensis* [41]; one sequence with 28% identity with the precursor of venom protein 29 and one sequence with 45% identity with the precursor of venom protein 302, both deduced from the transcriptome analysis of the venom gland of *Lychas mucronatus* [32]. We also found one sequence with 44% identity with the precursor of the orphan peptide AbOp5 deduced from the transcriptome analysis of *Androctonus bicolor* [66]; and one sequence with 49% identity with the precursor of Tbah02469 deduced from the transcriptome analysis of *T. bahiensis* [41]. Finally, two components had hits with 25% identity (both) with the precursor of venom allergen 5 deduced from the genome analysis of the spider *Stegodyphus mimosaroum* (only submitted to GenBank), and one component had hits with 55% identity with the precursor of putative scp tpx 1 ag5 pr1 deduced from the transcriptome analysis of the mite *Ixodes ricinus* [67].

Several transcripts putatively coding for precursors with an odd number of cysteines were found. Most of them contained a cysteine within the signal peptides (e.g., sdC14319_g1_i1). The presence of odd cysteines in signal peptide sequences has previously been observed, with the Gaussia luciferase (UniProt Q9BLZ2) signal peptide as a classic example. Since the signal peptide is cleaved by a signal peptidase upon translocation of the nascent secretory proteins to the endoplasmic reticulum, the odd cysteines play no roll in the folding or biological function of the mature secreted protein. We found a few transcripts potentially coding for proteins with odd cysteines within their mature sequence, two for a hyaluronidase (e.g., sdC14647_g1_i1) and three for CAP peptides (e.g., sdC3852_g1_i1). Proteins with an odd number of cysteines can potentially form dimers or link to other proteins. Whether this is the case for the here-reported transcripts remains to be demonstrated. The remaining deduced protein sequences with odd cysteine numbers (e.g., sdC14619_g1_i1) correspond to incomplete CDS, therefore the missing sequence most probably also contains an odd number of cysteines, which results in an even total.

### 2.3. Amino Acid Sequence Determination of Venom Components

The soluble fraction obtained from the venom was analyzed by LC-MS/MS, which resulted in the sequencing of 26 proteins/peptides with identity to translated transcript from the RNA-Seq (Table 3; Supplementary Table S2. Eight toxins (including four sodium channel toxins and two potassium channel toxins), two enzymes, four La1-like peptides, two CAP peptides, and six Non Disulfide Bridged Peptides were identified by mass spectrometry.

### 2.4. Phylogenetic Affinities of the Calcins, Scorpines, La1-Like Peptides and Potassium Channel κ Toxins Found in the Transcriptomic Analysis of the Venom Gland of S. donensis

#### 2.4.1. Calcins

**Figure 8:**
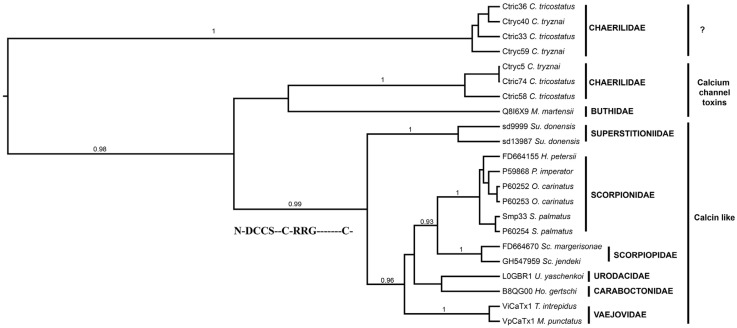
Phylogenetic tree obtained from the Bayesian analysis of 22 sequences of putative and confirmed calcins from 14 scorpion species belonging to 12 genera and eight families selected from the InterPro database and the available literature. The originally reported names are used (or the UniProt or GenBank accession codes for those lacking a name), followed by the scorpion species (see Supplementary Table S3). Posterior probabilities higher than 0.76 are indicated above the branches.

The result from the phylogenetic analysis of 22 putative and confirmed calcins reported for 14 scorpion species showed the presence of two “groups” of calcins. One clade grouped most of the sequences (18), whereas the other grouped only four sequences from two species, members of one scorpion family (Chaerilidae). The two components found in the *S. donensis* transcriptome analysis were grouped together and were related to the remaining of the calcins of the non-buthid scorpions in a clade named Calcin-like 1 (Figure 8). This clade is a sister group to the one formed by the toxin BmCa1 (Q8I6X9) from the venom of *Mesobuthus martensii* (Buthidae) plus three putative calcins found in two species from the genus *Chaerilus* (Chaerilidae). This pattern reflects the phylogenetic relationships between the two scorpion’s parvorders: Buthoida (Buthidae and Chaerilidae) and Iuroida (Caraboctonidae, Scorpionidae, Scorpiopidae, Superstitioniidae, Urodacidae and Vaejovidae). One sequence motif was found in the signal peptide of 14 of the sequences grouped in Calcin-like 1 (see Supplementary Figure S1).

The sequences grouped within the Calcin-like 1 clade have the following motif: NNDCCSKKCKRRGTNPEKRCR with an E-value of 1.2 × 10^−136^ (but see also Supplementary Figure S2). Calcins from species of the Scorpionidae, Scorpiopidae and Vaejovidae families were recovered as monophyletic. The calcins from scorpions of family Scorpionidae are the most studied (e.g., impercalcin or imperatoxin); and since the six sequences from this family were recovered as monophyletic, we further found two sequence motifs for that clade (see Supplementary Figure S3).

Four (out of seven) sequences from putative calcium channel toxins deduced from the transcriptome analysis of two species of *Chaerilus* were recovered as monophyletic, with high posterior probabilities. They constitute a sister group to the rest of the calcins mentioned before (Figure 8). These sequences were used as queries in BLAST searches against the UniProt database, and none had hits with calcium channel toxins, not even with scorpion calcins or with other known calcins from arthropods or mollusks. Therefore, these sequences should not be considered as true scorpion calcins unless proven otherwise (by experimental validation).

Our results were partially consistent with those presented earlier [3]. Both analyses recovered the differences between buthid and non-buthid calcins. However, the main difference between these two analyses (except for the terminal sequences used) is the fact that maurocalcin (P60254) from *Scorpio palmatus* was not grouped with the rest of the scorpionid calcins in [3]; whereas in our analysis it was recovered with the highest support within scorpionid calcins (Figure 8). Calcins have not been (or were not) found for species of the families Bothriuridae (one species in genus *Cercophonius*), Hemiscorpidae (genus *Hemiscorpius*; however, no transcriptomic data are available yet) and Hormuridae (genera *Opisthacanthus* and *Liochelis*; with no transcriptomic data available yet). However, they have been found in 12 genera of eight families; and they appear to be more common in the venom of scorpions of family Scorpionidae (thus far, four of the nine genera in the family have been studied). This suggests that calcins might be ubiquitous to scorpion venoms.

#### 2.4.2. Scorpines

**Figure 9:**
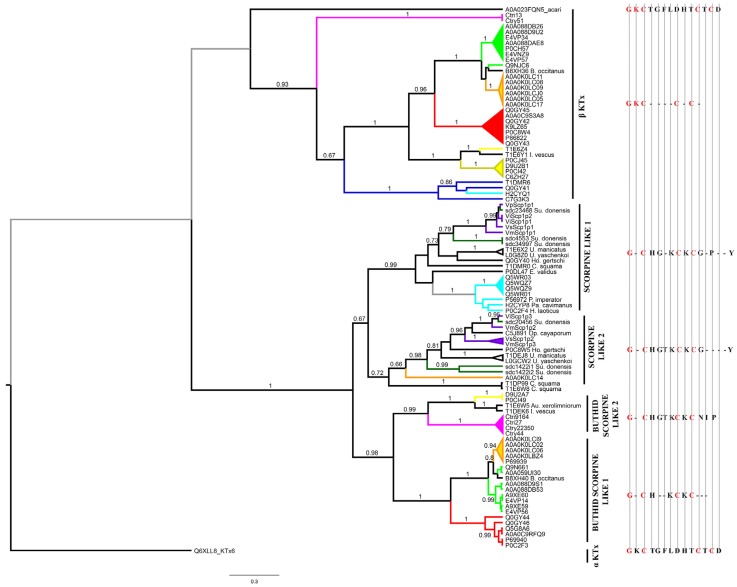
Phylogenetic tree obtained from the Bayesian analysis of 62 sequences of scorpines and putative scorpines, plus 34 sequences of βKtx or putative βKtx from 34 scorpion species of 22 genera and 10 families, and one sequence as outgroup (αKTx), selected from the InterPro database and the available literature. Terminal names are composed of UniProt or GenBank accession codes and the name of the scorpion species, except for those named as in their original publications (see Supplementary Table S4). Posterior probabilities higher than 0.65 are indicated above/below branches. Clades in red represent sequences from species of genus *Tityus*; in light green sequences from species of genus *Mesobuthus*; in orange sequences from species of genus *Androctonus*; in magenta, sequences from species of genus *Chaerilus*; in yellow sequences from species of genus *Lychas*; in purple sequences from species of family Vaejovidae; in light blue sequences from species of family Scorpionidae; in dark green sequences from *S. donensis*, and in dark blue sequences from several non buthid families.

The Bayesian phylogenetic analysis of 62 sequences of scorpines and putative scorpines, 34 sequences of β-like KTx; plus two outgroup sequences (one scorpion αKTx, and one “long chain scorpion toxin” from a mite) recovered scorpines grouped into a single clade with 100% support. The β-like KTx clade had high posterior probabilities and it was split into three groups: one with two chaerilid β-like KTx sequences, another with only buthid β-like KTx sequences, and the last one composed by four β-like KTx sequences from non-buthid scorpion species, including the potassium channel toxin Hge βKTx (Q0GY41, Figure 9; and Supplementary Figure S4). Within the clade of scorpines, we recovered two major clades: (a) one including non-buthid species plus only one buthid sequence, with 67% of support, and subdivided further into two more clades (Scorpine-like 1 and 2) (Figure 9); and (b) the other clade including only buthid species, with 98% of support, and subdivided further into two more clades (Buthid scopine-like 1 and 2) (Figure 9). The scorpine sequences deduced in the present contribution from *S. donensis* were included in the Scorpine-like 1 and 2 clades.

The clade Scorpine-like 1, supported by high posterior probabilities, includes the original scorpine (isolated from the *P. imperator* venom; see Supplementary Figure S5). Figure 9 shows that all scorpine sequences obtained from scorpions belonging to the same family were recovered as monophyletic, except for family Superstitioniidae, since one of our sequences (sdc23468_g1_i1) was recovered within the Vaejovidae family clade. With lower posterior probabilities, the clade named Scorpine-like 2 (see its motif in the Supplementary Figure S6) included one scorpine from a buthid species (AbKTx1 isolated from the venom of *Androctonus bicolor*) and 12 sequences from non-buthid scorpions. Like in Scorpine-like 1, one of our sequences (sdc20456_g1_i1) was grouped within Vaejovidae; and the rest of the scorpines were grouped accordingly to their familial hierarchy.

The last two clades (Buthid Scorpine-like 1 and 2; motif in the Supplementary Figure S7) were supported by high posterior probabilities and included scorpines from scorpions of parvorder Buthoida (Figure 9). Scorpines from species of genera *Androctonus*, *Chaerilus*, *Lychas* and *Tityus* were recovered as monophyletic supported by high posterior probabilities; but not those from genus *Mesobuthus* (Figure 9). As suggested by Santibáñez-López et al. [5], our results confirm that scorpines had an independent origin from the potassium channel α toxins.

#### 2.4.3. La1-Like Peptides

**Figure 10:**
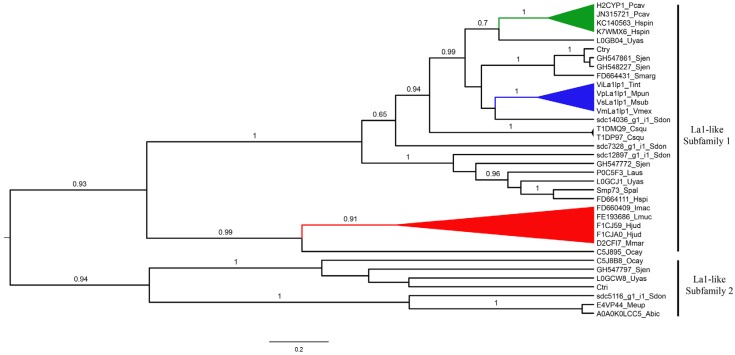
Phylogenetic tree obtained from the Bayesian analysis of 36 sequences of La1-like peptides or putative La1-like peptides from 23 scorpion species of 18 genera and nine families selected from the InterPro database and the available literature. Terminal names are composed of UniProt or GenBank accession codes and the name of the scorpion species, except for those named as in their original publications (see Supplementary Table S5). Posterior probabilities higher than 0.65 are indicated above branches. Colored clades indicate monophyletic groups of La1-like peptides from scorpions of families Buthidae (red), Scorpionidae (green) and Vaejovidae (blue).

Two major clades were recovered in the phylogenetic analysis of 36 La1-like sequences from 23 scorpion species (Figure 10): the La1-like clade subfamily 1 and subfamily 2. The La1-like subfamily 1 (La1.1) clade included 29 sequences with the motif IPVGQXKXDPXXCTLYKCXXXNNRXVLXKXTCA with an E-value of 1.8 × 10^−307^ (see also Supplementary Figure S8). It was further subdivided into two clades: (a) one with all buthid La1-like peptides (red clade in Figure 10), sister group to a putative secreted protein deduced from cDNA cloned from *O. cayaporum* (UniProt accession number C5J8B8); and (b) the other clade with 23 sequences from different scorpion families. This last clade can also be divided into two subgroups, one including the original La1 from *Li. australasiae* (P0C5F3) and five more sequences from scorpions of families Scorpionidae, Scorpiopidae, Superstitioniidae and Urodacidae. The other clade included La1-like peptides from scorpions of families Bothriuridae, Chaerilidae, Scorpionidae, Scorpiopidae, Superstitiionidae, Urodacidae and Vaejovidae.

The La1-like subfamily 2 clade included seven sequences of putative La1-like peptides from scorpions of six families (Figure 10) and the motif VTPVPPNCTLVRGRGSYPDCC with an E-value of 1.08 × 10^−28^ (but see Supplementary Figure S9). However, the internal relationships within this clade were not supported by high posterior probabilities (<50%), except for the clade with the two buthid SVWC-like peptides (100%). Three of the four sequences, deduced in the transcriptome analysis of the venom gland of *S. donensis* with identity with La1-like peptides, were found in the La1-like clade, but not forming a monophyletic group; whereas the other sequence was grouped within the SVWC-like peptides clade as sister group to the buthid sequences (Figure 10). These results suggest that the diversity of these peptides is greater in some families, such as Hormuridae (genus *Liocheles*) and in Superstitioniidae.

#### 2.4.4. Potassium Channel κ Toxins (κKTx)

**Figure 11:**
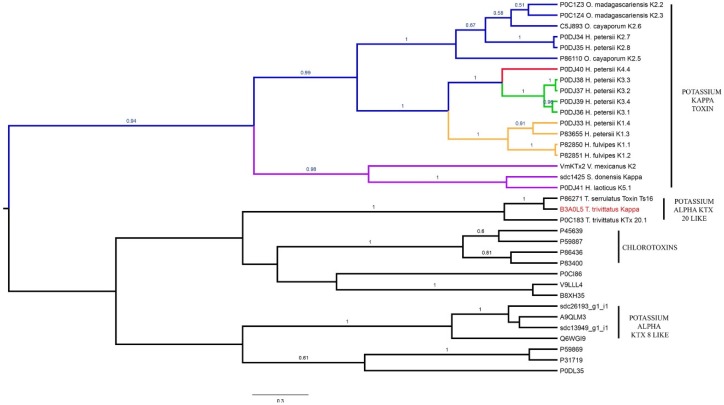
Phylogenetic tree obtained from the Bayesian analysis of 20 sequences of potassium channel κ toxins (κKTxs) from eight scorpion species of four genera and three families; and 12 sequences of potassium channel α toxins and chlorotoxins as outgroup, selected from the InterPro database and available literature (see Supplementary Table S6). Posterior probabilities are indicated above branches. Colored clades indicate the monophyletic subfamilies proposed earlier: subfamily 1 (orange); subfamily 2 (blue); subfamily 3 (green); subfamily 4 (red); and subfamily 5 (purple). The name in red shows κ buthitoxin.

The Bayesian phylogenetic analysis of 20 κKTx from eight scorpion species and 12 sequences of chlorotoxins and αKTx from 11 species showed the presence of the five subfamilies (Figure 11) as proposed earlier [42]. Subfamily 1 had the motif GHGCYRSCWREGNDEETCK with an E-value of 4.4 × 10^−18^ (Supplementary Figure S10); and included four κKTx deduced from cDNA cloned from the venom of *He. petersii*.

Subfamily 2 had the motif DPCVEVCLQHTGNVKECEEAC with an E-value 3.9 × 10^−34^; and included two κKTx from two scorpionid species of genus *Heterometrus* (*He. petersii* and *He. fulvipes*; Supplementary Figure S11). Subfamilies 3 and 4 were recovered as sister groups, while subfamily 4 is only represented by the κKTx 4.1 deduced from the transcriptome analysis of *H. petersii* (P0DJ40). Subfamily 3 included four sequences also deduced from the analysis of the same scorpion species.

Finally, subfamily 5 had the motif MKVLPLLFVFLIVCVMLPTEASCTQ (in the signal peptide), but with a low E-value (9 × 10^−5^). This subfamily was represented by three sequences including the κKTx found in *S. donensis*, one κKTx from the species *V. mexicanus* and κKTx 5.1 from *H. laoticus* (the original member of this subfamily; Supplementary Figure S12).

Our results differed from those presented earlier (e.g., [5]), since we recovered the potassium channel κ toxins family as monophyletic. In the previous analysis [5], subfamily 5 was not recovered as closely related to the κKTxs but to chlorotoxins. Of course, the main scope of that study was to explore the status of the current classification and the phylogenetic affinities of the CSαβ toxins. Both analyses recovered κ buthiotoxin Tt2b, Ts28 and Toxin Ts16 as a monophyletic group, but it was not closely related to the rest of the κKTx. This is not surprising since Saucedo et al. [68] mentioned that κ BUTX Tt2b and Ts16 toxins have the CSαβ motif, but adopt a CSαα motif in solution. These authors elegantly discussed the 3D structure of these toxins. However, they hesitated to establish a new subfamily for these toxins. Our results are in accordance with their proposed relationship between these three toxins, but not with their proposed relationship between these toxins and the κKTxs. We lack evidence to establish a new subfamily for these toxins, so we decided to include them in the αKTx 20 subfamily [69].

## 3. Conclusions

The 135 transcripts annotated here highlight the differences between buthid and non-buthid scorpion venoms. The annotated transcripts constitute just a small fraction of the whole assembled transcriptome. This reflects the lack of thorough knowledge on the toxinology and cellular biology of scorpions and prompts for a deeper investigation in the area. Future research on different unexplored scorpion families would contribute to the understanding on the diversity of venom components and their evolution. The discovery of calcins, scorpines and La1-like peptides in different scorpion families suggests the ubiquitous presence of these components in scorpion venoms. Some of them (e.g., calcins and La1-like peptides) can contribute to our knowledge on venom evolution when more families are sampled. Given that our results on the phylogenetic affinities of calcins partially mirror the phylogenetics of some scorpion families, we recommend the use of these peptides in generic phylogenetic reconstructions.

## 4. Materials and Methods

### 4.1. Biological Material

Scorpion specimens were collected in Ensenada Baja California, Mexico on August 2015. Permit for collection was issued by SEMARNAT (Scientific Permit FAUT-0175 granted to Oscar Francke, see acknowledgments). They were maintained in plastic boxes with water and fed with crickets. The venom was extracted by electric stimulation. Two specimens were sacrificed to process the telsons, and the other two were deposited at the Colección Nacional de Arácnidos in the Instituto de Biología of the Universidad Nacional Autónoma de México, in Mexico City.

### 4.2. Molecular Mass Determination and Protein Identification

The venom obtained was solubilized in water and centrifuged 10,000 g for 10 min. The soluble fraction of the total scorpion venom was desalted using ZipTip C18 (Millipore, Billerica, MA, USA) and 5 µg of the desalted material was then analyzed by applying LC-MS/MS. The LC was performed using an Accela HPLC from Thermo Scientific (San Jose, CA, USA) at a flow rate of 500 nL/min (splitter 19:1). The RP-C18 column (75 μm ID × 100 mm) used was constructed in house and a gradient ranging from 5% to 70% solvent B over 120 min was applied. Solvent A was made up of 0.1% acetic acid/water, and solvent B consisted of 0.1% acetic acid/acetonitrile. Eluted venom components were electrosprayed with a nano-electrospray at a voltage of 2.4-kV into an LTQ-Orbitrap Velos mass spectrometer (Thermo Fisher Scientific, Waltham, MA, USA). MS data acquisition was carried out automatically, using a data acquisition method specifically devised for molecular mass determinations. The data were automatically deconvoluted using the Xcalibur software for each 10 min run. Adducts formed by different combinations of Na and K as wells as single and double methionine oxidations were eliminated of the mass list.

Protein identification of the components present in the crude venom was performed using tryptic digestion in solution and subsequently analysis by LC-MS. First, 45 µg of the soluble venom was reduced with DTT 10 mM, alkylated with iodoacetamide 55 mM and desalted using ZipTip C18 (Millipore, Billerica, MA, USA). Secondly, the tryptic digestion was applied into an LTQ-Orbitrap Velos mass spectrometer (Thermo Fisher Scientific) using the same gradient described previously for molecular mass determination. MS data acquisition was accomplished automatically, using a method specifically devised for “de novo” sequencing. MS data were acquired with 30,000 resolutions in the FT-Orbitrap analyzer in the positive ion mode and only the five most intense doubly and triply charged ions were selected for dissociation by CID (Collision Induced Dissociation) and HCD (High-energy Collision Dissociation). Dynamic exclusion of 60 s was enabled and a pre-exclusion of 30 s was applied. Finally, the normalized collision energy was set at 35 arbitrary units, with an activation Q of 0.250 and an activation time of 30 ms for both CID and HCD. MS data were search against the cDNA database obtained from previous transcriptomic analysis using the Protein Discoverer 1.4 program (Thermo-Fisher Co., San Jose, CA, USA).

### 4.3. RNA Extraction, RNA-Seq and Venom Gland Transcriptome Assembly

The telson from two specimens were dissected under RNAse-free conditions and pooled into a single tube. RNA was isolated using the SV Total RNA Isolation System (Promega, Madison, WI, USA) following the protocol provided by the manufacturer. Briefly, the dissected telsons were manually macerated to homogeneity with a Kontes microtube pellet pestle rod (Daigger, Vernon Hills, IL, USA) in a 1.5 mL microtube with the provided RNA Lysis Buffer. After dilution with the RNA Dilution Buffer the sample was heated at 70 °C for 3 min, then centrifuged to discard all cellular debris. The cleared lysate was mixed with 95% ethanol and transferred to one of the spin baskets supplied by the kit. After washing with the RNA Wash Solution, the sample was treated with the provided DNAse for 15 min and then washed twice with the RNA Wash Solution. After centrifugation, total RNA was recovered in Nuclease-Free Water. The RNA was quantitated with a Nanodrop 1000 (Thermo Scientific) and its integrity was confirmed using a 2100 Bioanalyzer (Agilent Technologies, Santa Clara, CA, USA).

A complementary DNA (cDNA) library was constructed from the total RNA obtained, using the Illumina TruSeq Stranded mRNA Sample Preparation Kit, following the protocol provided by the supplier. Automated DNA sequencing was performed at the Massive DNA Sequencing Facility in the Institute of Biotechnology (Cuernavaca, Mexico) with a Genome Analyzer IIx (Illumina, San Diego, CA, USA), using a 72 bp paired-end sequencing scheme over cDNA fragments ranging in size of 200–400 bp. Each library consisted of two fastq files (forward and reverse reads), from which the adaptors were clipped-off. The quality of cleaned raw reads was assessed by means of the FastQC program (http://www.bioinformatics.bbsrc.ac.uk/projects/fastqc/).

This Transcriptome Shotgun Assembly project has been deposited at DDBJ/EMBL/GenBank under the accession GFCD00000000. The version described in this paper is the first version, GFCD10000000.

The short reads were assembled into contigs in a de novo fashion with the Trinity software (v. 2.0.3), using the standard protocol [70], executing the strand-specific parameter and normalizing the reads. Basic statistics as the number of “genes”, transcripts and contigs were obtained by running the TrinityStats.pl script.

The assembled contigs were used as queries to perform a BLAST analysis against UniProt (http://www.uniprot.org). They were then annotated with Trinotate (https://trinotate.github.io/, [73]). The signal peptides were predicted using the SignalP 4.0 server (http://www.cdbs.dtu.dk/services/SignalP/) and the propeptides were determined with the ProP 1.0 server (http://www.cbs.dtu.dk/services/ProP/). The theoretical molecular weights of the putative mature peptides were obtained using the ProtParam server (http://web.expasy.org/protparam).

### 4.4. Multiple Sequence Alignments, Phylogenetic Analysis and Motif Search

Multiple sequence alignments of the sequences found and their similar sequences were obtained using the online version of MAFFT ver. 7.0 [71] (http://mafft.cbrc.jp/alignment/server/). Alignments were edited in Jalview [72] and Adobe Illustrator CS6. We retrieved 188 sequences from the InterProt database (http://www.ebi.ac.uk/interpro/), GenBank database (http://www.ncbi.nlm.nih.gov/) or the available literature corresponding to: (a) 22 calcins from 14 scorpion species in 12 genera and 8 families; (b) 96 scorpines from 34 species in 22 genera and 10 families, the sequence of Toxin 38 from a mite, one sequence of αKTx from one scorpion species as outgroup; (c) 36 La1-like peptides from 23 species in 18 genera and 9 families; and (d) 20 sequences of potassium channel κ toxins from 8 scorpion species in 4 genera and 3 families, plus 12 sequences of potassium channel α toxins and chlorotoxins from nine scorpion species as outgroups.

We constructed four matrices (aligned each separately with MAFFT ver. 7.0 [71]) as follows: (a) 22 calcins with a length of 90 amino acids; (b) 98 terminal scorpines and outgroups with a length of 134 amino acids; (c) 36 terminal-La1 like peptides with a length of 161 amino acids; and (d) 35 terminal sequences including κKTx, Chlorotoxins and αKTx with a length of 102 amino acids. The best fitting model of protein evolution was selected using ProtTest 3 [73,74] and the Akaike information criterion on the basis of which the following models were selected: (a) for the calcin matrix the JTT + I + G was chosen; (b) for the scorpine matrix the LG + I + G was chosen; (c) and for the La1-like peptides and the κKTxs matrices the WAG + I + G was chosen.

The phylogenetic analyses were conducted under the Bayesian inference using the algorithm implemented in the software BEAST 1.8 [75]. These analyses comprised 30 million generations, sampling every 1000 generations, and those sampled before stationarity discarded using the burn-in option in TreeAnotator (included in BEAST 1.8 software package). The resulting topologies were edited with FigTree 1.4 (http://tree.bio.ed.ac.uk/software/figtree/) and Adobe Illustrator Cs6.

Considering monophyletic clades with high posterior probabilities (>76%), sets of their included sequences of amino acids were selected to establish motifs using the Multiple Em for Motif Elicitation server (MEME 4.10.0 at http://meme-suite.org/tools/meme [76]) and the Motif Alignment & Search Tool (MAST) to determine whether the selected motif was a unique signature or not.

The prediction of the signal peptide, propeptide and mature peptide was performed using the ArachnoServer (http://www.arachnoserver.org/spiderP.html); SignalP 4.1 Server (http://www.cbs.dtu.dk/services/SignalP/), and ProP 1.0 Server (http://www.cbs.dtu.dk/services/ProP/).

## Figures and Tables

**Table 1:** Venom studies, cDNA and/or transcriptomic analysis from the current scorpion families recognized by [1,2,3,4,5,6,7,8,9,10]. * Denotes manuscript submitted for publication, now under revision.

Family	Venom Studies Available	cDNA or Transcriptome Analysis Available
Akravidae	No	No
Bothriuridae	No	Yes
Buthidae	Yes	Yes
Caraboctonidae	Yes	Yes
Chactidae	No	No
Chaerilidae	Yes	Yes
Diplocentridae	No	No
Euscorpiidae	Under revision *	Under revision *
Hemiscorpiidae	Yes	No
Heteroscorpionidae	No	No
Hormuridae	Yes	Yes
Iuridae	No	No
Pseudochactidae	No	No
Scorpionidae	Yes	Yes
Scorpiopidae	Yes	Yes
Superstitioniidae	This study	This study
Troglotayoscidae	No	No
Typhlochactidae	No	No
Urodacidae	Yes	Yes
Vaejovidae	Yes	Yes

**Table 2:** Presence of calcins, scorpines, La1 like peptides and potassium channel κ toxins in the eleven scorpion families with venomic or transcriptomic studies as indicated in Table 1.

Family	Calcins	Scorpines	La1-Like Peptides	Potassium Channel κ Toxins
Bothriuridae	No	Yes	Yes	No
Buthidae	Yes	Yes	Yes	No
Caraboctonidae	Yes	Yes	Yes	No
Chaerilidae	Yes	Yes	Yes	No
Hemiscorpiidae	No	No	No	No
Hormuridae	No	Yes	Yes	Yes
Scorpionidae	Yes	Yes	Yes	Yes
Scorpiopidae	Yes	Yes	Yes	No
Superstitioniidae	This study	This study	This study	This study
Urodacidae	Yes	Yes	Yes	No
Vaejovidae	Yes	Yes	Yes	Yes

**Table 3:** Transcripts coding for components present in the venom, as validated by mass spectrometry. Abbreviations: # Pep. = number of sequenced peptide fragments corresponding to a given transcript; Seq. Cov. = sequence coverage; NaTx = sodium channel toxins; KTx = potassium channel toxins; Sc = Scorpine-like peptides; NDBP = Non-Disulfide-Bridged Peptide.

Peptide Type	Transcript	Score	# Unique Pep.	Seq. Cov.	MW (kDa)	Protein/Accession
NaTx	sdc14462_g1_i1	257.51	7	91.43%	11.7	Lipolysis activating peptide 1 alpha chain/93140443
	sdc14462_g2_i2	29.7	2	29.81%	11.8	Birtoxin/20137305
	sdc15193_g1_i1	20.23	1	85.71%	7.1	Toxin Cll7/31376362
	sdc14462_g1_i2	118.64	1	78.10%	11.8	Altitoxin/116241245
KTx	sdc13949_g1_i1	27.98	2	65.38%	5.5	Toxin KTx 8/159146538
	sdc14273_g1_i2	58.09	1	76.32%	4.1	KTx 6.7/74838004
Sc	sdc14222_g4_i1	296.09	3	86.90%	9.3	Hg scorpine like 2/224493299
	sdc14222_g4_i2	153.55	3	76.47%	9.2	Hg scorpine like 2/224493299
NDBP	sdc12606_g1_i1	59.38	2	50.00%	4	Vejovine/325515699
	sdc14106_g1_i1	81.77	2	81.82%	2.5	Amp1/932534523
	sdc28695_g1_i1	45.7	2	77.27%	2.4	Amp2/932534537
	sdc4010_g1_i1	15.61	1	100.00%	5.2	Heterin 1/485896696
	sdc13544_g1_i1	83.11	1	86.79%	6.4	CYLIP Cer 2/522802549
	sdc14358_g5_i1	207.52	1	62.50%	1.8	Amphiphatic peptide CT2/384382524
	sdc14358_g12_i1	211.79	1	52.38%	2.3	CYLIP Cer 2/522802549
	sdc6540_g1_i1	161.85	1	52.38%	2.3	CYLIP Uro 3/522802596
La1-like	sdc13004_g1_i1	111.52	4	92.68%	9.1	Putative secreted protein/240247657
	sdc14036_g1_i1	162.81	2	100.00%	8.4	La1 like protein 15/430802826
	sdc12897_g1_i1	50.14	2	74.32%	7.9	La1 like protein 13/430802824
	sdc14589_g1_i1	53.02	1	90.24%	8.8	Toxin like protein 14/430802832
Enzymes	sdc14619_g1_i3	310.92	9	87.73%	67.7	Neprilysin 1/567441193
	sdc14393_g1_i1	70.24	4	76.13%	26.4	Phospholipase A2/218546750
	sdc14212_g1_i1	90.57	3	74.19%	24.1	Phospholipase A2/218546750
	sdc14619_g1_i1	42.67	1	78.17%	83.3	Neprilysin 1/567441193
CAP	sdc3852_g1_i1	97.67	3	71.20%	43.1	CAP-Uro-1/522802590
	sdc13900_g1_i1	87.21	2	54.10%	38	CAP-Iso-1/522802633

## Supplementary Materials

The following are available online at www.mdpi.com/2072-6651/8/12/367/s1, Supplementary Tables S1–S6; Supplementary Figures S1–S12.
