# Supplementary material for: Venom Gland Transcriptomic and Proteomic Analyses of the Enigmatic Scorpion Superstitionia donensis (Scorpiones: Superstitioniidae), with Insights on the Evolution of Its Venom Components
Source: Toxins (Basel). 2016 Dec 9;8(12):367. doi: 10.3390/toxins8120367 (PMC5198561; doi:10.3390/toxins8120367)
Supplement: Supplementary file 1 [file toxins-08-00367-s001.pdf]

Supplementary Materials: Venom Gland Transcriptomic and Proteomic Analyses of the Enigmatic Scorpion *Superstitionia donensis* (Scorpiones: Superstitioniidae), with Insights on the Evolution of Its Venom Components

Carlos E. Santibáñez-López, Jimena I. Cid-Uribe, Cesar V. F. Batista, Ernesto Ortiz and Lourival D. Possani

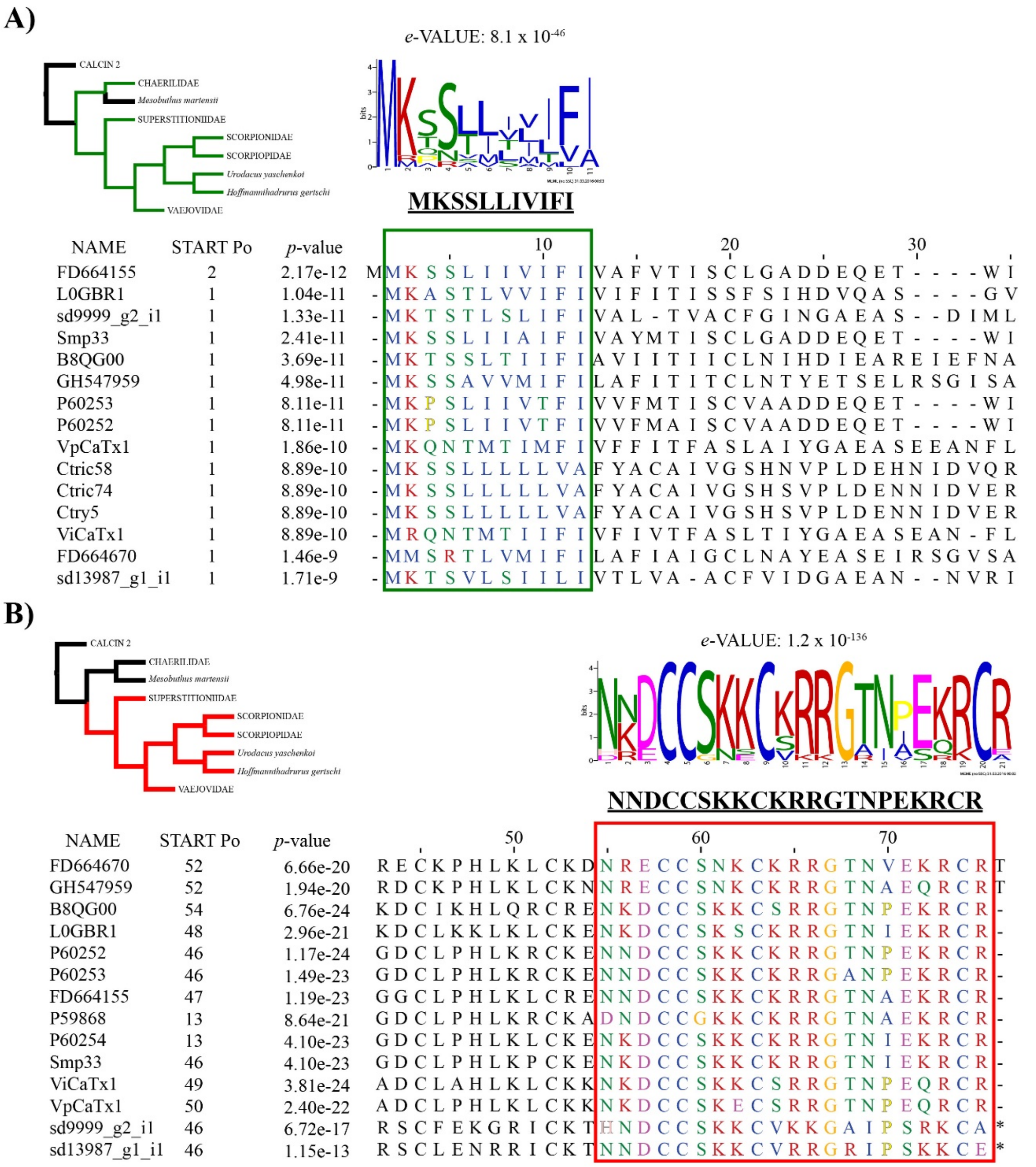

Figure S1. Amino acid sequence alignment of true calcins (Figure 8), retrieved from MAFFT's analysis; showing the two motifs found with MEME, along with a cladogram with branches with the Motifs colored. *e*-values above MEME's logo. Consensus sequence underlined. (**A**) Motif 1, missing in the Buthidae branch; (**B**) Motif 2, missing in the Chaerilidae and Buthidae clade.

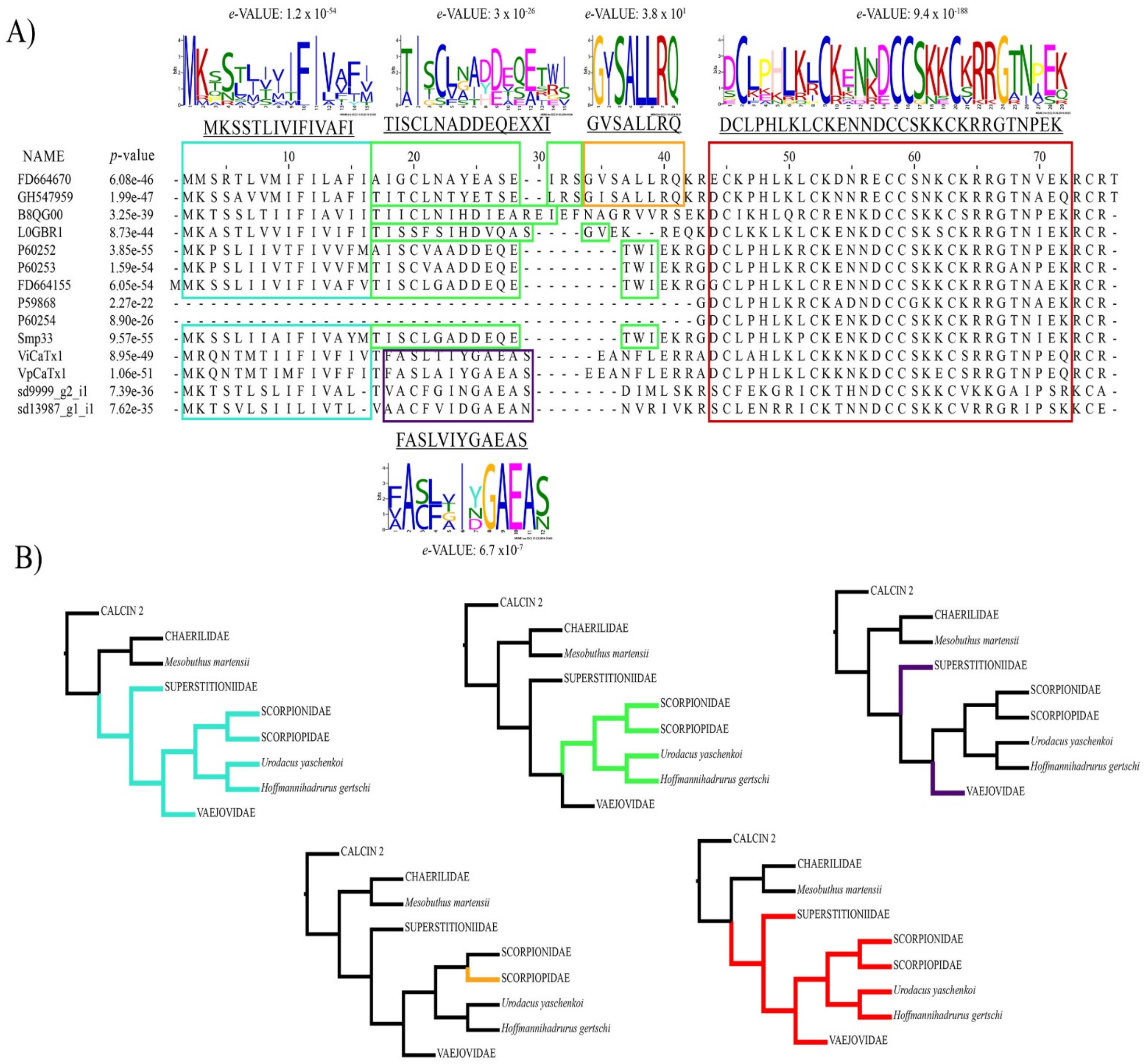

Figure S2. (**A**) Amino acid sequence alignment of true calcins (Figure 8), retrieved from MAFFT's analysis, from scorpion species of the Parvorder Iuroida showing five plausible motifs found with MEME; *e*-values above MEME's logo; consensus sequence underlined; (**B**) Cladograms colored accordingly to the colors of the motifs where they are present.

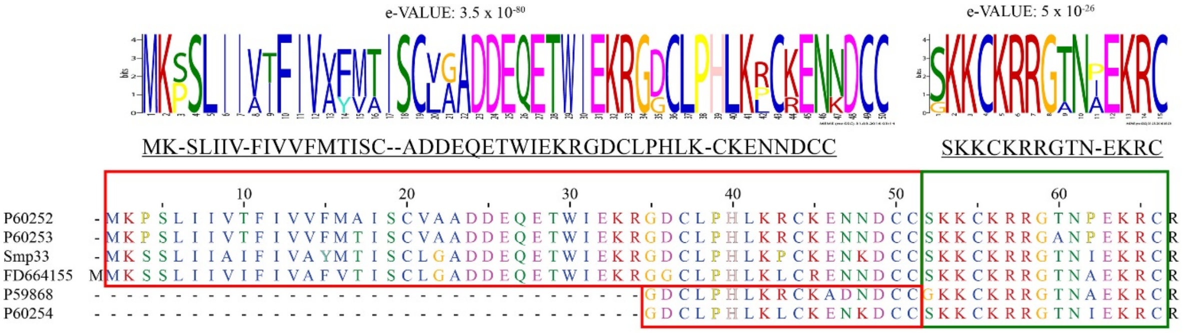

Figure S3. Motifs found in the amino acid sequences alignment of the six Calcins (Figure 8), retrieved from MAFFT's analysis, found in the venom of scorpion species of family Scorpionidae. *e*-values above MEME's logo. Consensus sequence underlined.

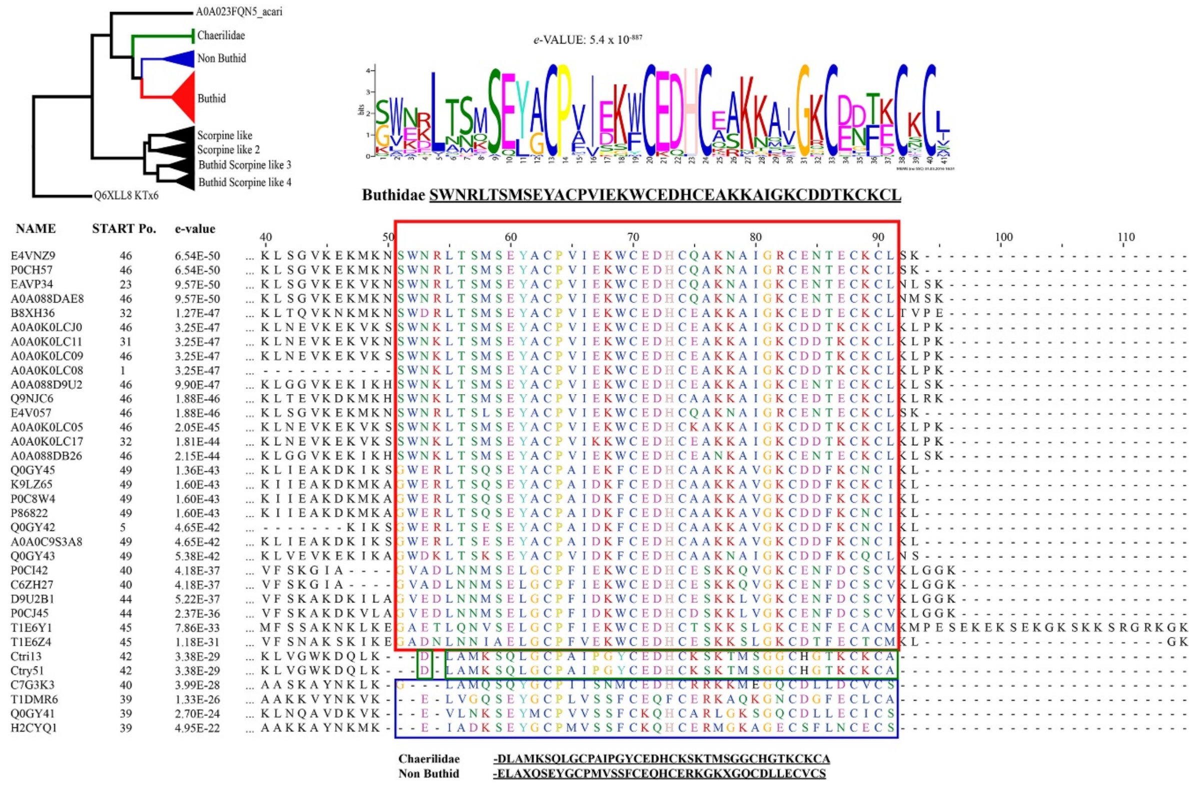

Figure S4. Amino acid sequence alignment of βKTx used in this study (Figure 9), retrieved from MAFFT's analysis, showing the motif found with MEME, along with a cladogram with branches with the Motif colored. *e*-value above MEME's logo. Consensus sequence underlined.

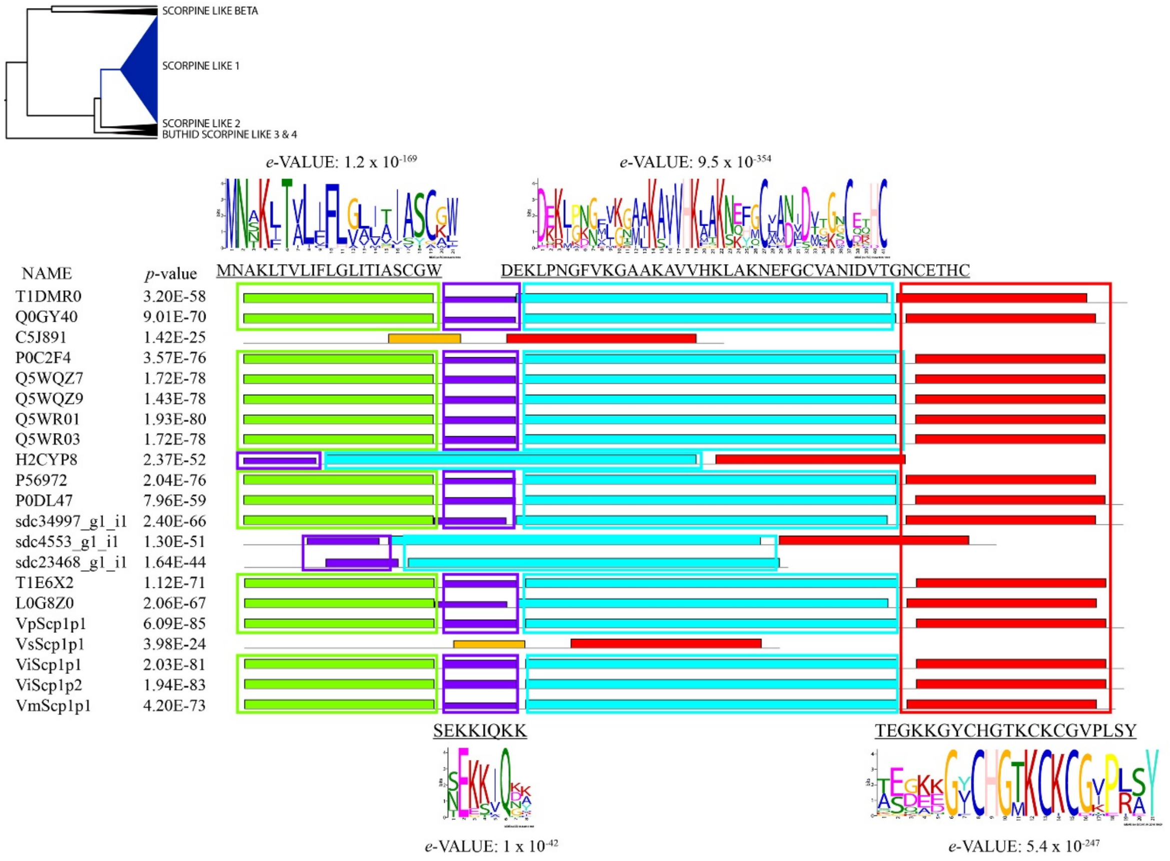

Figure S5. Amino acid sequence alignment of Scorpine like 1 (Figure 9), retrieved from MEME's analysis, showing the motifs found with MEME. Consensus sequence underlined.

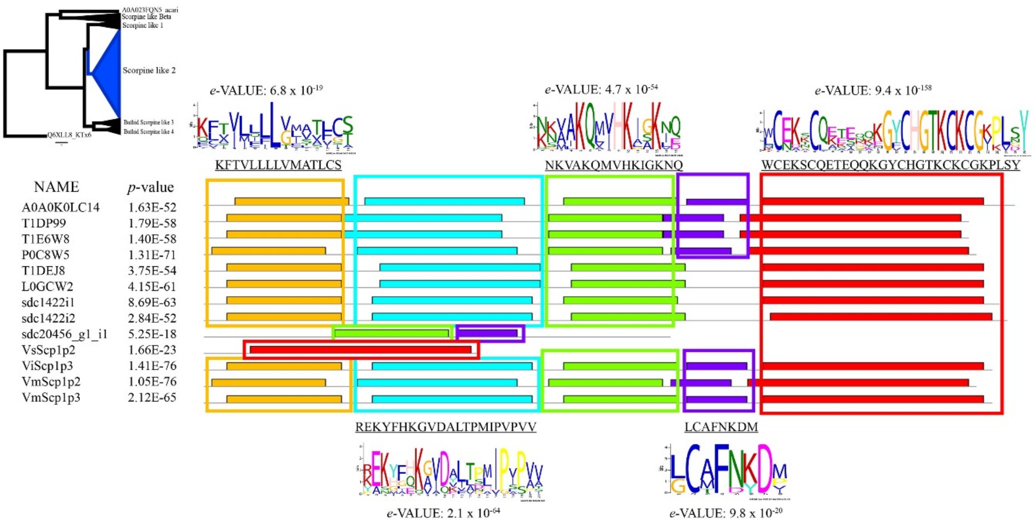

Figure S6. Amino acid sequence alignment of Scorpine like 2 (Figure 9) retrieved from MEME's analysis, showing the motifs found with MEME. Consensus sequence underlined.

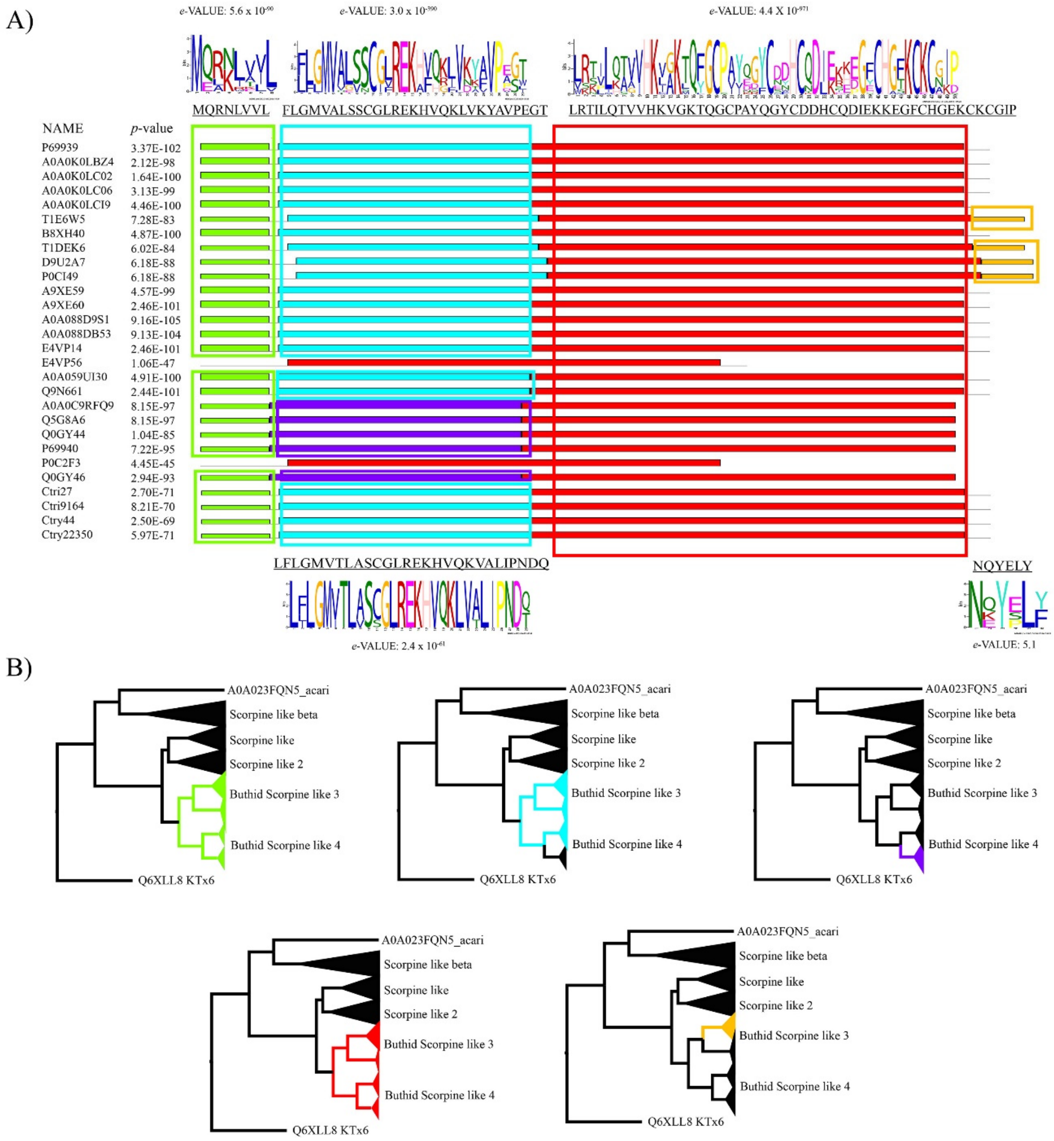

Figure S7. (**A**) Amino acid sequence alignment of Buthid Scorpine like clades 1-2 (Figure 9), retrieved from MEME's analysis, showing the motifs found with MEME. Consensus sequence underlined; (**B**) Cladograms colored accordingly to the colors of the Motifs showing their presence in each clade.

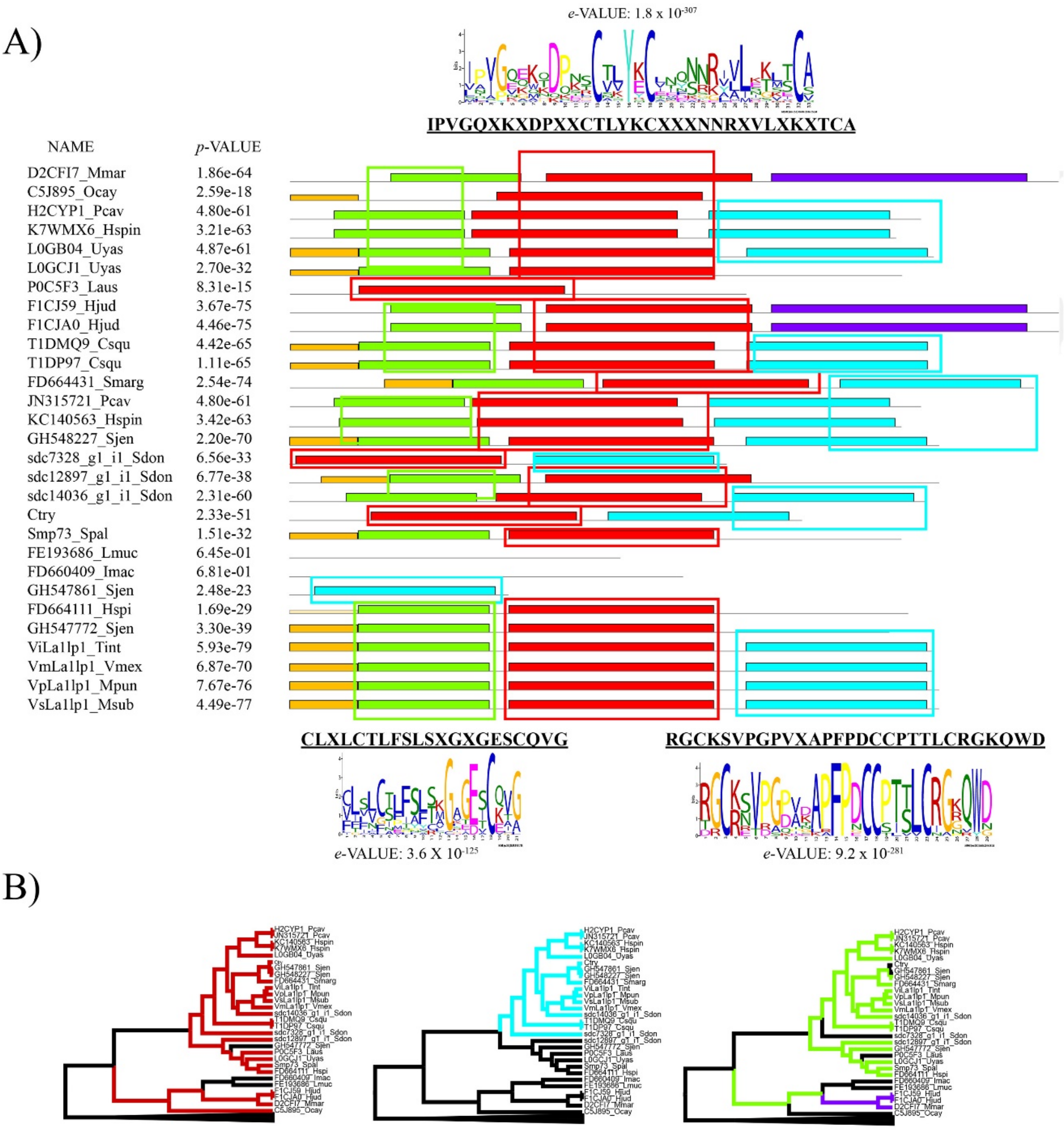

Figure S8. (**A**) Amino acid sequence alignment of La1 like peptide (Figure 10), retrieved from MEME's analysis, showing the motifs found with MEME. Consensus sequence underlined; (**B**) Cladograms colored accordingly to the colors of the Motifs showing their presence in each clade.

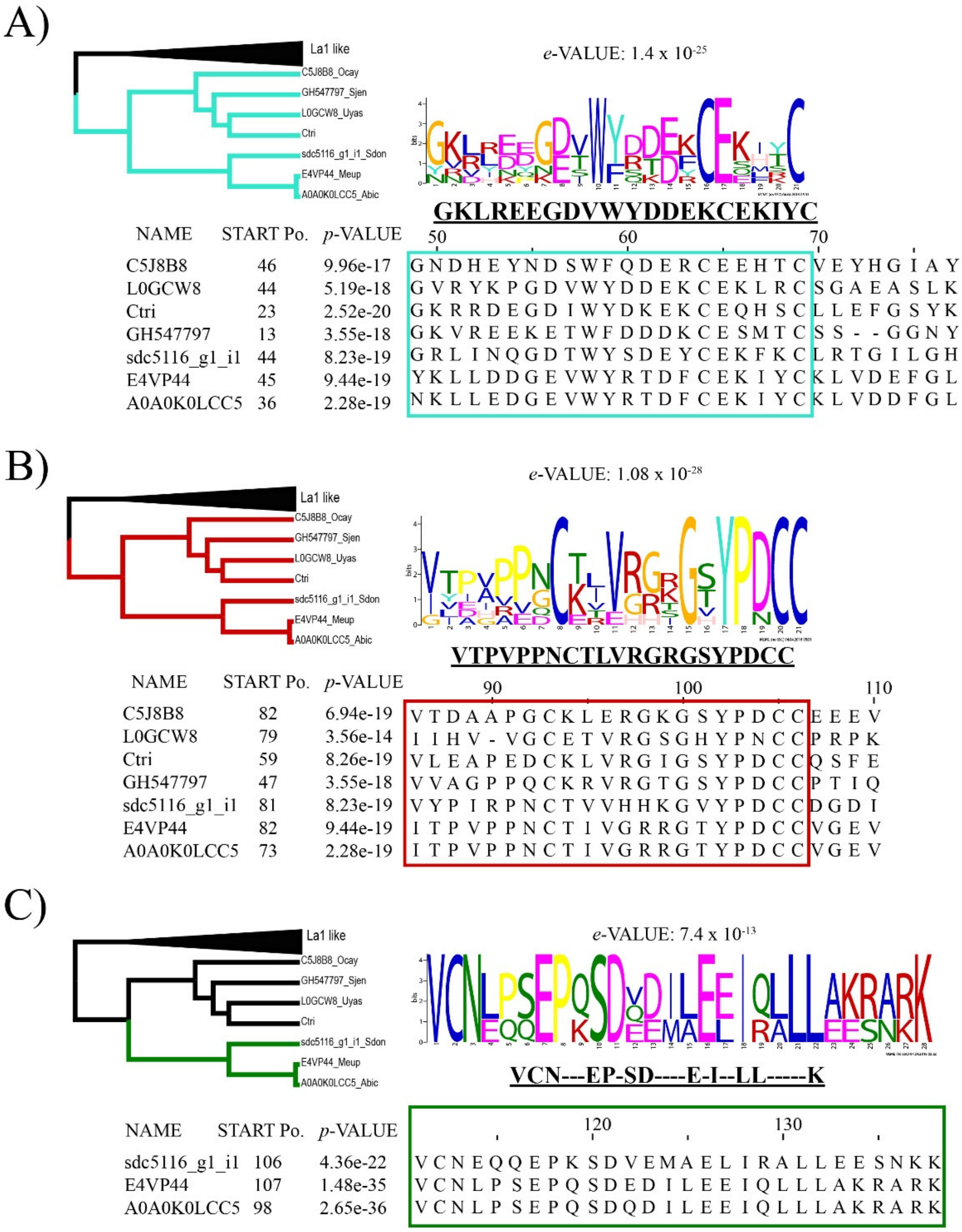

Figure S9. Amino acid sequence alignment of SVWC Scorpion like peptides (Figure 10), retrieved from MAFFT's analysis, showing the motifs found with MEME. Consensus sequence underlined. Cladograms colored accordingly to the colors of the Motifs showing their presence in each clade. (**A**,**B**) Motif for all SVWC Scorpion like peptides; (**C**) Motif only for three sequences including two buthid SVWC Scorpion like peptides and one superstitioniid.

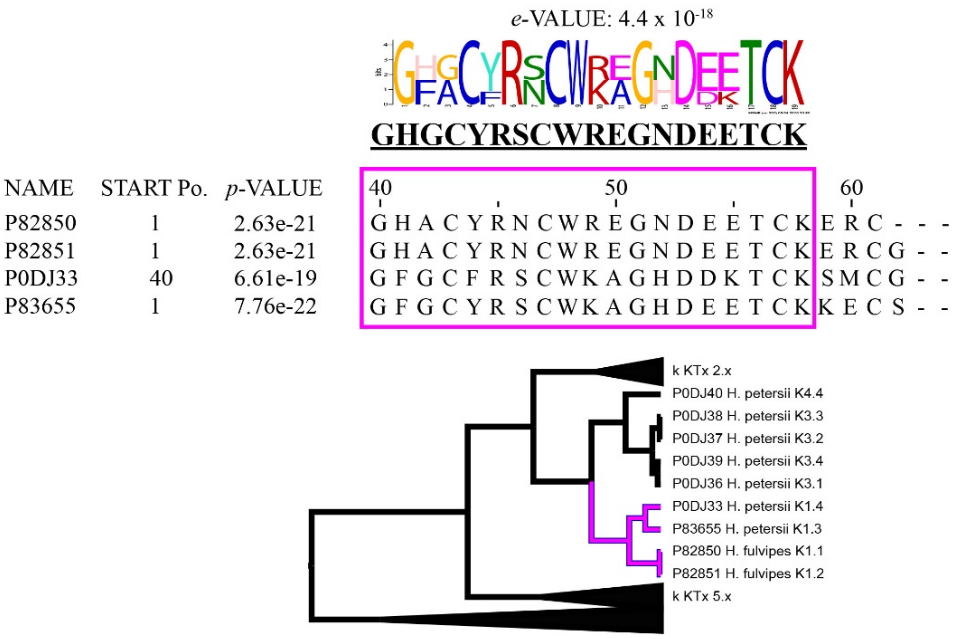

Figure S10. Amino acid sequence alignment of κKTx subfamily 1 (Figure 11), retrieved from MAFFT's analysis, showing the motif found with MEME. Consensus sequence underlined. E-value above MEME's logo. Cladogram colored accordingly to the presence of the motif.

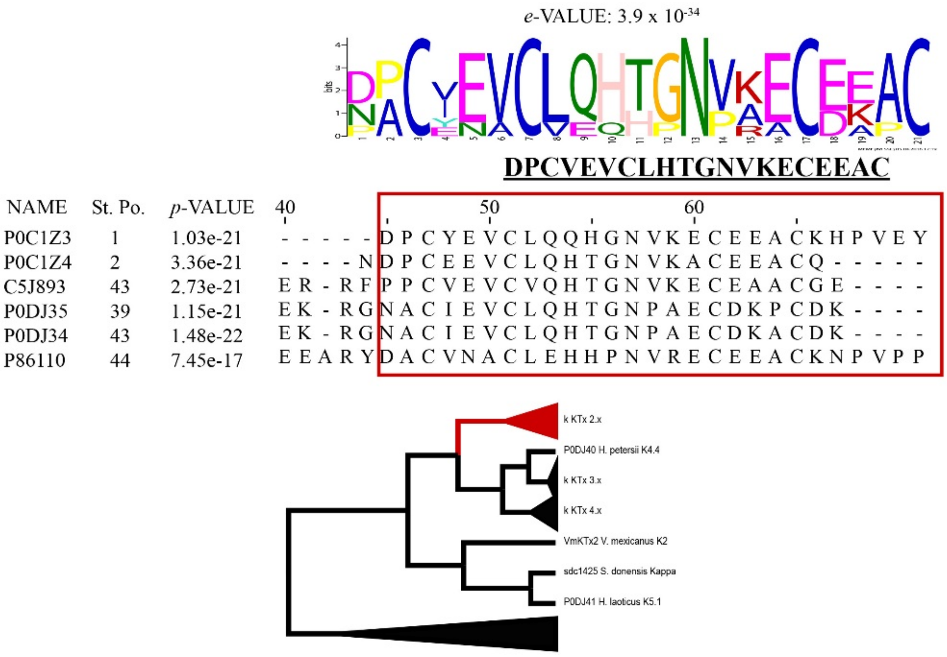

Figure S11. Amino acid sequence alignment of κKTx subfamily 2 (Figure 11), retrieved from MAFFT's analysis, showing the motif found with MEME. Consensus sequence underlined. E-value above MEME's logo. Cladogram colored accordingly to the presence of the motif.

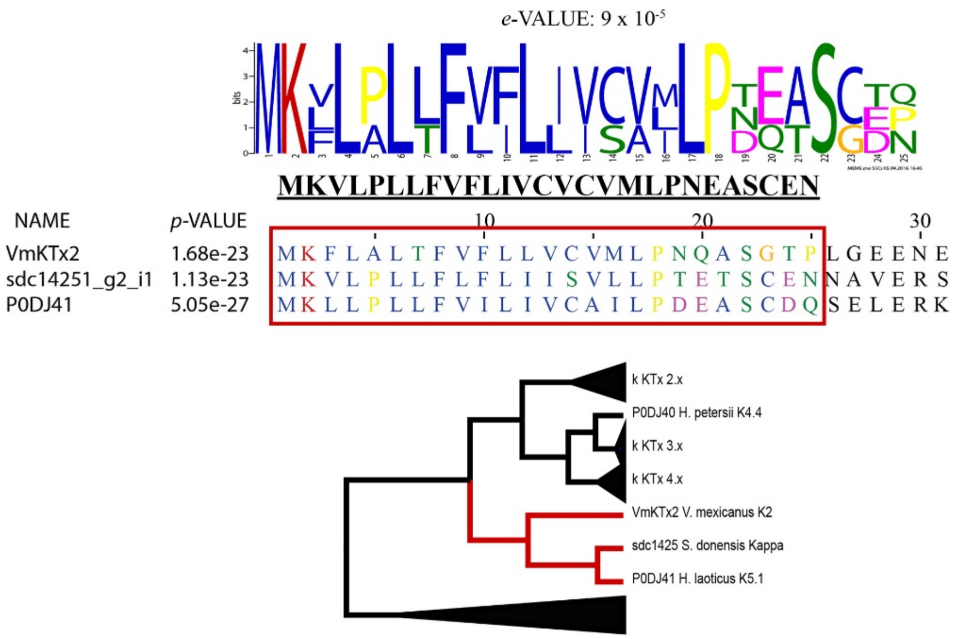

Figure S12. Amino acid sequence alignment of κKTx subfamily 2 (Figure 11), retrieved from MAFFT's analysis, showing the motif found with MEME. Consensus sequence underlined. E-value above MEME's logo. Cladogram colored accordingly to the presence of the motif.

Table S1. Unique sequences encoded by 135 transcripts of the *Superstitionia donensis* venom transcriptome. In underlined: Signal peptide. In bold: propeptide cut. In brackets, the GI number from GenBank accession number; in asterisk terminal stop codon.

	**TOXINS**	
	SODIUM CHANNEL TOXINS	
sdc14319_g1_i1	MNNCTCFILCLVALIYEFGNAQGKDGYPLNDMGNTIFCVMVQSGNEKCEKNCKERGGHGYCYALRCYCKGMK NNVKIWE *	Csab Cer 2 (522802561)
sdc14462_g1_i1	MKTAFLSIVVFLLMLHLDTVLLQIYDGDLYKKGGSTIPCLKHGGDSYCVDVCAKHGAHGGSCYSIPSRCWCEGLDI RKHGGYPKNEKGDYVWCGLPGGENKECEDVCRKQEAGYGYCYDRYCWCEGK *	Lipolysis activating peptide 1 alpha chain (93140443)
sdc14462_g1_i2	MKTAFLSIVVFLLMLHLDTVLLQIYDGDLYTKDGSIPCLKHGQDKYCKRICAEHGAHGGSCHGLPSRCWCEGLDI RKHGGYPKNEKGDYVWCGLPGGENKDCQDVCKKKGSGYGYCYDRYCWCEAPQ *	Altitoxin (116241245)
sdc14462_g2_i2	MMKTAVLSIVGILLLLNLDIVVLQMYDGYIYDDDGTHYPCRRIIGHKYCEELCKDVGAGEGWCKAFPSGCFCRGL DIRKHGGYPRDKNGDWIWCGLPGGKNKECENVCTEQGAGYGYCYDRYCWCEAP *	Birtoxin (20137305)
sdc15193_g1_i1	MKRLALLILGFIVLDVIDAEEEGGYPLREGEPAVYYNCAEGGTEYCIKVCRKIDAGYGYCYGLIRTCYCEGLTITKH RGYPN	Toxin Cll7 (31376362)
sdc16570_g1_i1	MGKVALRIVGIIIIFHIDRIFSYEGGYPVLINGADLYYGCSPSNDYCNKLCLAMDAGEGYCYIDNCFCKGLHVSKHA AYPKTDGGDYIWCYDNRQNNRYCEQVCKRYKGGPGICALNYHVCYCWDPVLEMGEFTIPESLYE *	Toxin TdNa8 (294863151)
sdc21236_g1_i1	MEGVSTLLIIVTIAISRLSSVASYEGYLIVKGTDNYYNCDIRGNGNVYCRQICHKFDAGDGHCQGIYSQSCWCKNLR ISKHGGYPKQSNGKLYSCWKDEYCEGICKQHDAAYGYCYASSCYCE *	Neurotoxin LmNaTx30 (317412111)
sdc10528_g1_i1	MKSVVLSIIWVLVLLLQDTVVLQKYDGYLYHKNGSYYQCYRATVSNCPSVCHKYGAYGGECHGTPAGCFCKGLEI KKHGGYPMENGEYVWCGGDDEMCDDVCKRQEAESGYCYSSRYCWCVPP *	Toxin Pg8 (187763642)
	POTASSIUM CHANNEL TOXINS	
sdc10141_g1_i1	MNKTLIFSMLLLITIMMTIGDADGERCSSSTYCNSYCWKKAKCTRGKCINKECKCYNCGRGD *	Potassium channel alpha KTx 6.10 (74838001)
sdc13949_g1_i1	MRAPTGGCPFSDALCANYCKKNKFGKGGKCDGPKCKCSIKMAPFDAKVPLTE *	Toxin KTx 8 (159146538)
sdc26193_g1_i1	MNKVHCTILLVVLMVFAVSVLPIEGVPTGGCPSDALCAKYCKSNKYGSTGKCDNTNCKCSVG *	Toxin KTx 8 (159146538)
sdc14251_g2_i1	MKVLPLLFLFLIISVLLPTETSCENNAVERSGDSFAELSRSIVKRSCKRVCSGNRRSKQCMQKCKNGR *	Potassium channel alpha KTx 5.1 (384950677)
sdc14273_g1_i1	MRKLLIVFLVLTVLEMAIVPQVDASGEPCSTSKQCTAPCRAGGGSHGKCMNRRCKCYG *	Potassium channel toxin alpha KTx 12.5 (302425088)
sdc14273_g1_i2	MRKFLIVLLVLTVLEMAIVPQVDASGEEPPIWPCRTSKQCTDPCRAGGGSHGKCMNGKCRCY *	Potassium channel toxin alpha KTx 6.7 (74838004)
sdc13860_g1_i2	MTKLFIVLLVSTVIAMTIVPKVDASGEKCRNSAQCKDICRAETGGQGRCMNSKCKCFVG *	Potassium channel toxin alpha KTx 6.7

		(74838004)
sdc13860_g1_i1	MTKLFIVLLVSTVIAMTIVPQVDASVGRCTASKQCTAHCRMRGESHGECENRRCKCY	Potassium channel toxin alpha KTx 6.10 (74838001)
sdc9772_g1_i1	MLLVITFMMAFDEINADGKKCLVSSECHDYCWKGNKCSRGKCINKRCKCYNCRG *	Potassium channel toxin (585636477)
sdc9772_g1_i2	MKKAIIFNMLLVITFMMAFDEINADGKKCLVSSECHDYCWKGNKCSRGKCINKRCKCYNCRG *	Potassium channel toxin (585636477)
sdc13973_g1_i2	MKAVLIIILVLAVLEVAIGVPQAYAPGVRCRNNRICQEVCPRSTKCINGKCRCYKG *	Tbah02745 (757181004)
	SCORPINE-LIKE PEPTIDES	
sdc34997_g1_i1	MNAKLTILFFLVLVSIASAGLTEKKVQGYLDKKLKDGMVKTALKSIVHKFTKSQYGCAADMDVTGNCQKHCQEA EQADGICHGMKCKCGVPRAYRK *	Hge scorpine (121953382)
sdc2871_g1_i1	DVLTPMIAVPVVGGILNKVAKQMLHKIGKLDSPCIFGID *	Hg scorpine like 2 (224493299)
sdc14222_g4_i1	MQIKLVVILFLGFATLSTGGFIKEKHFHKAIDVLTPMIPVPVVGGIVNKVAKQMVHKIGKLDTPCIFGIDKKGNCEK TCQETTHQKGYCHGTKCKCGKPLNYK *	Hg scorpine like 2 (224493299)
sdc14222_g4_i2	MQIKLTVLLLLGCVTIIAAGILKEKHFQKAIDEFVPMIPVPPSVSGALQKAAKQMVHKIAKLDSSCILGHDRGGKC NKGCQETVQQIGYCHGTKCKCGVPLGYR *	Hg scorpine like 2 (224493299)
sdc20456_g1_i1	VDALTPLIPVPVVGGIVNKVAKQMIHKIGKIQSLCAFNKDMAGLCEKKCQETEKLKGYCHG	Hg scorpine like 2 (224493299)
sdc4553_g1_i1	SIASAGLTEKKVQGYLDKKLKDGMVKTALKSIVHKFTKSQYGCAADMDVTGNCQKHCQEAEQADGICHGMKC KCGVPRAYRK *	Antimicrobial peptide scorpine like 1 (430802820)
sdc23468_g1_i1	ITIASCAWISEKKIQDAIDRKLPNGLVKNAIKAVVHKAAKNKHGCLADFDVGGGCEQHCR	Csab Uro 4 (522802580)
	CALCIN	
sdc9999_g2_i1	MKTSTLSLIFIVALTVACFGINGAEASDIMLSKRSCFEKGRICKTHNDCCSKKCVKKGAIPSRKCA *	Ca like 20 (430802828)
sdc13987_g1_i1	MKTSVLSIILIVTLVAACFVIDGAEANNVRIVKRSCLENRRICKTNNDCCSKKCVRRGRIPSKKCE *	Opicalcin 1 (37539456)
	SPIDER TOXIN	
sdc21328_g1_i1	MKLLAIAFIGCLLLLLVNEARSEGGDSMIRVARQARRCIPKYRSCDHNKSGCCDNASCRCNLFGTNCKCQRKGIF QG *	U8 agatoxin Ao1a (675369705)
	NON DISULFIDE BRIDGE PEPTIDES	
sdc30354_g1_i1	MNAKVFLAVFIVAMFVTDQAEAGFWKNVWNSDIAKNLRNKAVNWVQEKIGAPQAAKLDEFLNSLYYR *	Antimicrobial peptide Con22 (430802818)

sdc19033_g1_i1	VTDEAEALWGFLAKMASKVLPSLFSGGKK**R** *	Con10 (240247615)
sdc24668_g1_i1	VVLLHLISQSEALWGALLGLGSTLLQKLGK**R**GAQNLDQFDDIFEPELSEADIRYLQDLLR *	Amphiphatic peptide CT1 (384382522)
sdc36542_g1_i1	AHQHLVFTNSLYKMKTQLVVLIVALVLMQLFAQSEAFWGALLNAASSFLGK**R**	Amphiphatic peptide CT1 (384382522)
sdc12611_g2_i1	VAMLFLQFVSQSDAFLKGIFDTVSKWIGK**R**GLKNLDQYNDLFDGEISDADIKFLLDLMR *	Amphipathic peptide CT2 (384382528)
sdc8130_g1_i1	MYSGSDAEGYWGKLWSGDKSAASSIQGK**R**GLKNKDQYEDFYAPDLTAADLKLL	Amphiphatic peptide CT2 (403399470)
sdc14358_g5_i1	MKTQFVILIVALVLMQMFAESEAIFGAIWNGIKSLFGK**R**GLRDLDLDDNMFDDMYEPELSAADLKMLQDLFR *	Amphiphatic peptide CT2 (384382524)
sdc14106_g1_i1	MKLITLMPVFLCLLIVVDYCQSFPFFLASLIPSAINLVKKIGK**R**DADFQRYVDLKRRDLDLDELMSRLSEY *	Amp1 (932534523)
sdc28695_g1_i1	LTMQFKKALLVIFISYLLVTDEAEAFWGFLAKMAGKVLPSLLSSGK**K**DKRNREIEDFYDPNQRQ *	Amp2 (932534537)
sdc14244_g1_i1	MQFKKALLVIFISYLLVTDEAEAFWGFLAKMAGKVLPSLLSSGK**K**DKRKREIEDFYDPYQRQLDLENLLNQLDLN *	Amp2 (932534537)
sdc9431_g1_i1	TMQFKKALLVMFIAYLLVAHEVEAFWGALAKVATSVLPSLFSKRSSLRNVRK**R**EVGNFFDPYQKDLDLDDLFAQL DKY *	Amp2 (932534537)
sdc10966_g1_i1	MKTQLVVLIVALVFVQLFSGSDASFWGNLWGGMKSVVGSFLGK**R**GLKN	CYLIP Uro 1 (522802600)
sdc6540_g1_i1	MKSQLVVMIVALVFMQMFSESEAGFWGNVWEGIKSVGKNLLGK**R**GLRNMDQFDDLYEPDLSPAD	CYLIP Uro 3 (522802596)
sdc8996_g2_i1	MQMFSESAGFWANVWKGIKSIGKNLLGK**R**GLRNMDQFDD	CYLIP Uro 3 (522802596)
sdc13329_g1_i1	MKTQLVFLIVALVLVQMFSGSDAEGFWGKLWSGVKSAASSILGK**R**GLKNIDQYDDFYEPDLSAADLKLLQELFR *	CYLIP Uro 3 (522802596)
sdc10003_g1_i1	MKSQLVVLIVALVFMQMFSESEAGFWGNVWEGIKSVGKNLLGK**R**GLRNMDQFDDLYEPDLSPADLKFLQELLR *	CYLIP Cer 2 (522802549)
sdc13462_g1_i1	MKTQFLVLIVALVFTQIFSEAEAAGFWGKLWEGVKSVGKSLLGK**R**GLRNVDQFDDLYEPDLSPADLKFLQELLR *	CYLIP Cer 2 (522802549)
sdc13544_g1_i1	MKTQLVILAVALVYMQVFTESDAYFWTYTFPKHLFTRTPSPSLLGKSKLRNWDQFNDLYESDLSSADLKFLQKLLR *	CYLIP Cer 2 (522802549)
sdc14358_g12_i1	MKTQFVILIVALVFMQMFSESEAGFWGDVWSGIKTVGKNLLGK**R**GLKKLDQYDDMYEPDLDAADLEFLKELLR *	CYLIP Cer 2

		(522802549)
sdc13245_g2_i1	MKTQFVILTIALVLVQMFSESEAEFHSKVWGDLVKSLLGK**R**ELRSFDQFDDLYE	CYLIP Cer 3 (522802547)
sdc4010_g1_i1	MNAKVFLVVFIVAMLVTDQAEAGFWKNIWNSDIVKNLRNKAVNWVKEKVGAPQVAKLDEFLDSVYNS *	Heterin 1 (485896696)
sdc15372_g1_i1	EILLDPLLLMQMYAQSEAILGANWNGIKTLLGK**R**SL	IsCT2 (22213544)
sdc16828_g1_i1	KTQFVILFVALVLMQLFAESEAFFGAIWNGLKSLFGK**R**	IsCT2 (22213544)
sdc22496_g1_i1	MMTQFVFLIVALVFLQMFSLSEAGFWGVVWSGI *	IsCT2 (22213544)
sdc22251_g1_i1	MKTQFVVLIVALVFMQMISESEAGNWGDVWSG	NDBP 5.5 (152962885)
sdc37652_g1_i1	FVILIVDHVLMQMFEASEAIIGGHIKGKASLLGK**R**GLRDL	Peptide Hp1035 (347602456)
sdc39539_g1_i1	TQFVILIDDLVFKQMFSDSEDGFWGEVWRGIKSV	Peptide Hp1165 (614103404)
sdc12606_g1_i1	MNAKIFLVVLLVAAFMTEETEAGFFGKIWNSDFVKNLRNKAVKFISDKIGTNPPQAA**T**LDELLDALYS *	Vejovine (325515699)
sdc14749_g1_i1	MNAKTIFAIVLIGMLVTEQAEAGIWSTIKKYASKAWNSDIGKSLRNK	Vejovine (325515699)
sdc14209_g1_i1	MNTKTFLLVFLFALIVTEQAEAGLWGAIKSFAKNAWKSKLGRKLRTMASNALMPKPESVPMPVPVPEAVAEAVP SGSRLDFMYY *	Vejovine (325515699)
sdc4413_g1_i1	FAESEAIIGDIIGNLTAQFGKRSLRDLELDNDMYEPELSAADLKM *	Venom antimicrobial peptide 6 (149134048)
	**PROTEASE INHIBITOR**	
sdc12570_g1_i1	KMIVSCIFLILVLNAVFVAESSHHKSVNCLLPPKTGPCKGSFARYYFDIETRTCKAFIYGGCEPNTNNFAKRHHCEK RCKRFGARKYHGK *	Kunit type serine protease inhibitor (224493105)
sdc31500_g1_i1	MMFLLAFVLMTSLNVLVYSQDRCTLPPESGLCLAYFEKYYYDSNFRTCKMFVYGGCDGNDNRFDTEDACLAACA GSK *	HW11c39 (627610707)
sdc13156_g1_i1	FILWVPSISVATDLYNLQRSRMKFAIVALFVAITIIAQVTSQEPKCRKNEYYTTCGGCDGNCQEYVVPCTRMCHPPG CYCVENTVRGPNGDCIFTSECSGNEI *	Cysteine rich venom protein (366984639)
sdc13156_g2_i1	QRLKMKFTIVAVFVAITIIAQVTSQETNCPKNEYYRTCGNCDGNCRKHVVPCPRICHPAGCYCVGGTVRGPNGDC IFTNECP *	Cysteine rich venom protein (366984639)

sdc14810_g1_i1	MNALTGLTFTFILLCVLHFNSFSVKGDDNCEENEVYTKCGSICESCDDFITPRPCPEICFIGCACKEGYFRDTNEKCIP AEECKKQ *	Putative trypsin inhibitor like cysteine rich domain protein (604769033)
sdc3658_g1_i1	MLLQKFNMNIFLVVLTAITIAVGVRSQDQCWLNEYYTECGGCDGTCDEPLVACPYICHPAGCYCEYGTVRGPNG LCIAEEDCPKHK *	Putative salivary secreted serine protease inhibitor (366984585)
sdc6891_g1_i1	MKFILAPMLMAILVQVKSQDKPCGENEYYTECGGCDGTCAEPVVQCTKNCHPAGCYCQQGAVRGSDRKCILLE DC *	Putative salivary secreted serine protease inhibitor (366984585)
sdc13407_g1_i1	MKAALVLLFAAVFLNFQVKGHHVVGHEEGCASPDEVFDPCGPSCPPSCIGVIKPGTLCSTECVPGCYCRDGLVRT ARGTCVTPRACRNN *	Putative similar to chymotrypsin elastase inhibitor ixodidin (427777519)
sdc20776_g1_i1	MVTGTEVQCWWNESYTTCGGCDGTCAEPMPICHTICHEPGCYCNSGTVRGPDGFCIPLESCPNHK	Putative salivary secreted serine protease inhibitor (366984585)
sdc27767_g1_i1	MKFLLVAVFIVIIAIALVRSQEPQCGNNAFYMECGGCDGTCDEPLVACTMICHEPGCYCEWPNVRGPNGECIPRE QCP *	Putative salivary secreted serine protease inhibitor (366984585)
sdc12740_g1_i1	MGKFIILAVIVSMAVLVHAFPQAHDLFRCDDDEEMVTCLPPCPRSCSNLFNPRPCTVLLPTCRTGCSCKGGKIRGN DGRCVFPADCPRRN *	TSA Tbah02514 mRNA (757180944)
sdc1626_g1_i1	MKRNIVSGVFALAVLFIILDNCDAQYNSVGRNCTNPDQVFTECASACSLTCDDYVNPKNWCTLQCIIGCICRPGY AFQTTFGTRCVPISDCRRK *	Venom peptide SjAPI (528050044)
sdc3980_g1_i1	MKRNTILGVFALIVLFSILDKCEAEDLFEELSCKNPDEVFSQCFPICPLTCDDYVNPKPMCGLTDTCLFGCVCRRGY AFQNEIHSRCIPISDCKSG *	Venom peptide SjAPI (528050044)
sdc9177_g2_i1	ASPAAERCKKEGEEYDECGASCPETCENKGTPGPCFYVCLPGCLCK *	Venom peptide SjAPI (528050044)
sdc13359_g1_i1	MMKCNVALSIFVVIVLCSVFSGCVAQGNESRGRPCRSPNEEFTRCGTACPLTCQNYQNPPQVCTLQCVIGCVCKR GFIRENGVRSRCVRPQECRRY *	Venom peptide SjAPI (528050044)
sdc36886_g1_i1	GFIGSLCRNTAFCVLTILITCSVLESYMIQKNKSRNQCVLSGEEFTEKGSACPATCDNYQEVNKTCSVNSVIGCFCKT GLVRDVLNWRCVKPEEC *	Venom peptide SjAPI (528050044)
sdc13933_g2_i1	MNKYVFLYVFALLTFCCLHSSSGQRNGRPKFNCNRPGEEFRRCGTACPLTCDNYRSPPKSCTRQCVIGCACKNGL VKDRNGSCVPPSQCLR *	Venom peptide SjAPI 2 (528050045)
sdc39356_g1_i1	NPNEVFTGCSSACTLTCDHYVNPPELCSLQCIIGCVCR	Venom peptide SjAPI 2 (528050045)
	**ENZYMES**	

	METALLOPROTEASES	
sdc14592_g1_i1	MEQRSYNTITGGRSVDLLTKDEDVGFWAKRTRLEQALIVICSVLAIMAVCLIIAVVVISQKRNIDDVTSEAPPTTVTT<br>SKPIVEKVCETEICKLAADELKATINYSINPCEDFYSYVCSGWRRDNPIPESMVTYTKFGLLDEKIKNELIEVLPNIDK<br>YEDGDVRTKAKTFYTSCINTETREGKGVEGLKNVLKSVGGWPLLGENITEEYNWTDALVFSIRELGSSPIIAFAVGP<br>DAKNTSDNIVQIDQTSLSIGRNELLNSNKSDERVKEIMAAYKNLVKQSAMLLGSKKTEDQLDNDINDMVEFERTL<br>ATYTRSEEDRRDPNSLYKKMTIDGLQTGLNTEQIDILSFFNKIMANITELTNDTNIIVAEYDYLYQTCKLLGQNTTKP<br>ETIANYIGWRIVQNYGTETTENFTNTFLEFDKVTMGVEKSKPMNETCIDDANSLLDFALGNLFINHGFDVDTKTEI<br>ENMIAELKTAFGELLKSNKWMDNNIKEKALNKLNEMFPMVAYPPWLNDTDKLNEYYEKLGDIIEGSHLENVITIA<br>KFHTQKQLNKINKSYNRTEEWASAPAVVNAFYDPSANTITFPAGILQPP	Neprilysin 1<br>(567441193)
sdc14619_g1_i1	MEQKSYNTLNGGGSVYQLIKGKYAGFRVKITKLEQTLIVVCWVLVIVANVTSHKLTAEKVCDTEVCKLAADKLKD<br>TMNESINPCEDFYSYVCSGWKRDNPIPETMSALGNLHLLDENIKNELIEVLPKIVKYEEQDVRTKAKRFYTSCIDTE<br>TRQSKGVEALKAVLKSLGGWPLLGETIETGYDWTDALAYAKRELANHVIITFYVGPDDKNTSQNIIKINQASFGLK<br>RDNFINKNRSSEKVKKTMKAYKKLIKQSAKLLGSTKTEEELDSDVNDMIEFERTLAEFSRSDEEKKDLYSKYRKMTV<br>GRLQLELNTKQIDILLLLNKVMNNITKLTNNTEVIVMELDYLKQTCKLLEQNNTKTETIANYIGWIIVRGYGEHTT<br>KEFADAFFEFDKVTSGTAKAKPLNETCIKKANEYFRYALGNIFINNGFNDQTKTEIERMTVQLKTAFEELLQTNKW<br>MDKETKKESLNKLNEMIPMVAYPTWLNDTKKLNGYYDKLGEIIEGSHLENVINIVKFIIRTRLNKINKPFNRTEKW<br>GSGPAVANAFYSPNSNLIRLPAGILQPPFYQVGLPAALNFGGIGSIIGHEITHGFDNTGSQYDAQGNLKEWWTPEA<br>RSRFKNESKCFVEQYGSYKEPITGMNLNGNNTLGENIADNGGLRQAFKAYKSATSGIYSNMRLPGLDMTSDQLFF<br>VGFAYAWCADARKEHLELALKYDAHSPPPFRIRGVLSNFEEFAKAFKCP	Neprilysin 1<br>(567441193)
sdc14592_g3_i1	EQYGSYKDPITEMMLNGENTLGENIADNGGARESFKAYKSLTAVSEYNTRLPGLDLTADQLFFVGFSYVWCANER<br>KEYLEKAIQYDPHSPPSFRVRGVFSNLEEFSEAFGCPPNSTLNPKHKCLLW *	Neprilysin 1<br>(567441193)
sdc14619_g1_i3	MIMKGVAKVEQKSHNPINWGSNVYHLIKYAGFRVKRTPVEQTLIVIVCVLAIMADVVTSRKLITEKVCDTEVCKL<br>AADMLKATINDSINPCEDFHSHVCSGWKRDNPIPETMSALGNLHLLDENIKNELIEVLPKIVKYEEQDVRTKAKRF<br>YTSCIDTETRQSKGVEALKAVLKSLGGWPLLGETIKKGYDWTDALAYAKRELARNAIIKLYVGPDDKNTSQNIIKV<br>SDPSFGMKRDDFIDRSSEKVKRKIKAYKKLIKQSAKLLGSTKTEEELDSDINDMVEFERTLAKFSRTVEEKKDLYSKY<br>KKMTVGRLHLELNTEQIDILLLLNKVMNNITELTNSSEIIVMELDYLKRTCKLLEQNTTKTETIANYIGWTIVNGYG<br>RQTTKEFADAFFEFRKVSTGIVKATPLNITCIEKANYYFGLPLGNLFINNRFNVETKMEIENMVVQLKVAFEELLKT<br>NEWMDNETKKKSLNKLNEMITLVAYPSWLNDTKKLNGFYEKIGEIIEGSYLENTITIAKFHVQTSLGRINKPNNRT<br>KMWGNKIAGVEAYYDPKSNKIFLPAGVLQPPHYQVGLPPALNFGAIGSIIGHEITHGFDN	Neprilysin 1<br>(567441193)
sdc14619_g1_i5	MADVVTSHKLITEKVCDTEVCKLAADMLKATINDSINPCEDFHSHVCSGWKKDNPIPETMNLYGNLQMLNEKIK<br>NELMEVLPNIVKYEEEDVRTKVKRFYTSCIDIETRQAKGVEGLKAVLKSLGGWPLLGETIETGYDWTDALAYATSE<br>LAYNPIISFSVGPDDKNTSQNIIKVDEAKFGLKRDNLINTNRSNQKVRRIMKAYAKLIKQSAKLLGSVKTENELDN<br>DINDMIEFERTLAKFTRSKEEKRDLYSCYKKMTVDRLQLELNTEQIDILSLLNKVMKNITELTNNTKIIVKELDYLKQ<br>TCKLLEQNNTKTETIANYIGWIIVRGYGEHTTKEFADAFFEFDKVTSGTAKAKPLNETCIKKANEYFRYALGNIFIN<br>NGFNDQTKTEIERMTVQLKTAFEELLQTNKWMDKETKKESLNKLNEMIPLVAYPSWLNDTKKLNDFYEKLGNVI	Neprilysin 1<br>(567441193)

	QGSHLENTITIAKFHTKRKLDKINKPYNRTEMWGSGLVVANAYYAQKSNTIIFPAGILQPPFYQVGLPPALNFGGIGSSIGHEITHGFDNQGGMYDAQGNLKEWWTPEARNRFKYESKCFIEQYGSYREPKTGLMLNGKNTLGEN	
sdc14619_g1_i6	MEQKSYNTLNGGGSVYQLIKGKYAGFRVKITKLEQTLIVVCWVLVIVANVTSHKLTAEKVCDTEVCKLAADKLKDTMNESINPCEDFYSYVCSGWKRDNPIPETRAHYVNLLLVEDKIINELIEALQNAGKYEEEDVRTKVKRFYTSCIDIETRQAKGVEGLKAVLKSLGGWPLLGETIETGYDWTDALAYATSELAYNPIISFSVGPDDKNTSQNIIKVDEAKFGLKRDNLINTNRSNQKVRRIMKAYAKLIKQSAKLLGSVKTENELDNDINDMIEFERTLAKFTRSKEEKRDLYSCYKKMTVDRLQLELNTEQIDILSLLNKVMKNITELTNNTKIIVKELDYLKQTCKLLEQNNTKTETIANYIGWKTVKRYGRQTTKEFGNAFFEFDKVSSGIVKAKPLNETCIQNANGYFDFALANLFINNRFNVETKTEVETMVAQLKLAFVELLKTNKWMDNKTKKESLNKLNEMIPLVAYPSWLNDTKKLNDFYEKLGNVIQGSHLENTITIAKFHTKRKLDKINKPYNRTEMWGSGLVVANAYYAQKSNTIIFPAGILQPPFYQVGLPPALNFGGIGSSIGHEITHGFDNQGGMYDAQGNLKEWWTPEARNRFKYESKCFIEQYGSYKEPKTGVMLNGKNTLGENIADNGGLRQAFKAYKSHTSGNDRNMRLPGLDLTSDQLFFVGYAYVWCSNERKERLERDLKYGVHTPAFFRVRGTLSNSEEFA	Neprilysin 1 (567441193)
sdc13753_g1_i1	MLVFWTLTSIVLVFSVNGQFWDLRGDFCRTRKPQTCCPGRDDECTVPILDTLCYCDIFCNRTSGADCCPDFWPTCLGINPPGPIVTQQCFKDGRSYNVGESIKINCNKCTCRQRSPTNYDFVCEQNVCLVRPELIESINHGNYGWKASNYSFLWGKTLEEGIQYRLGTFKPTVSTMAMNEVKIKMRKELPENFDARQHWHNLIHPVRDQGDCGSSWAFSTTSLASDRLAIQSRGSMNMALSPQHLISCETRGQKGCDGGHLDRAWFYLKRHGVASEECYPYESGTSKENGRCAVRKPRGRHGHILCTNGQEDMVHKSTPAYRIGPQEDLIRQEIFNYGPVQATFKVYNDFFLYKGGVYRHTNLSDGKPEAYRLHGWHSVRIIGWGIDRTGRRPVKYWLCDNSWGRLWGEDGYFRIIRGEDECDIEMFIIGVWANTNSNNMNGIGH *	Putative cysteine proteinase ixodes scapularis cysteine proteinase (604813376)
sdc14484_g1_i1	MNATRKFLLLSICIVLASCNFSDLPGPYCRTR**R**PVDCCPGRDDSCAVPILGTLCYCDHFCNRTYGQDCCPDFLPHCLGITPPEPLVHRVCHHEGRTYQVGQTIRKNCNACTCRPTSATTFEFNNCEERVCLVRPELIDGINRGNYRWRASNYTFLWGKSLDEGIRLRLGTFQPTEQASRMTAIHQGKDPLPSSFDSRRKWPNLIQPVRDQGNCGSSWAFSTTAVASDRLGIQSQGKERLSLSPQHLVSCQNRAQRGCHGGHIDRAWWFLRKRGVASESCYPYESGETGHKGTCLAKLSPNGNEPRIKCVSGGREDILHYSTPPYRIGRKEEDIMHEIFTNGPVQATFQVKEDFFLYKSGVYHHTPVSAHLPGKYRRSGWHSVRIIGWGVDHSHGRPVNYWLCANSWGSRWGEDGYFRIIRGRNECDIENFVLATWGKRRTSNGRYYGRAGRRR**R**S *	Putative cysteine proteinase ixodes scapularis cysteine proteinase (604813376)
sdc4278_g1_i1	DRLPTFDVAVVITKLDMCRRQFNGGRCSRGTAGFAYVGGACVVNKRLEKVNSVAIIEDSGGFSGIIVAAHEVGHLLGCVHDGSQPPSYLGGPGATNCPWEDGYIM	Uncharacterized protein like *Tetranychus urticae* (no GI available)
	PHOSPHOLIPASES	
sdc12400_g1_i1	MALTFFVAVTMLLSLAYAHIAERELYVNFEPLPNQQDSWPVARAAIVTHRSEAGREFTECRMLGSIEELAREGRSLPEHLIKRTSKAEMDEFERICSAASEAERFMIVPGTKWCGPGNKAANESDLGWLAADKCCRAHDHCDSISQGKSKYGLKNEGEYTLLNCDCEKAFHKCLTETADSQNWITSKPTQGIRYTYFTLYKPKCYKVSCGGGRSAIESRKCSNLVATWKNSYLD *	Phospholipase A2 (218546750)

sdc14212_g1_i1	MTFVFLTAIIALLSLAYSHTTERELYVNFEPLPNQDDSWPAARAAIVSFRSEAGREFSECRMLNSVEELAREGINLPK<br>HMIKRASAEEMDDFERRCSRSADRERFMIAPGTKWCGPGNKAANYSDLGSLEADKCCRTHDHCDNIPKGKSKY<br>GLTNDGEYTLLNCNCDKAFDSCLQNAANKEANSVDKATTNAIKFAYFTVYAPKCYRLSCGGGRSDMEGRACAN<br>AVGTWKSSYLA *	Phospholipase A2<br>(218546750)
sdc14393_g1_i1	MVFISLIIFTTFVWIGSTQNIQKELYVNFEPLPGQKDSWPIARAAIVSFDKSSEARRTLPECRMLHSLEEIAREGNYFSE<br>RMIKRVSKEEMNTLERRCS**R**SSQEEERFSLTFIKWRDTKWCGPGNDAANETDFGLLEADKCCYAHDHCDSISSSES<br>KYGLKNDEHITLLHCDCEEAFDKCLQDTANKVNSDYQKEQTQQLRHFYFVTIKHRCYRLYCENKRDNSTCRGYW<br>RKDYKDEDYD *	Phospholipase A2<br>(218546750)
sdc14634_g4_i1	MGMQNLIFVITFTLVSMAWAQSSEKELYLNFEPLPGQRDGWPVVRAVRMLYQKRSEGGRELRTFDGCQILDSITEI<br>SREAYRMPRHSMKRISKEEMKSFEGRCERSGEVERTFLGTKWCGAGNTSTSYSDLGTLNNIDSCCRDHDHCDSIAA<br>GETKYGLKNKGENSLMNCDCEVAFAACLDEVPSKTYWFLSKATEKLSIPAIKKFYFDWYGNSCYNLTCSSGRSLSN<br>NECTNPVAEYNGPTGFFNPLFNG *	Phospholipase A2<br>(218546750)
sdc14634_g4_i2	MQNLIFVVTFTLVSVAWAQSSEKELYLNFEPLPGQRDGWPVVRAVRMLYQKRSEGGRELRTFDGCQILDSITEISRE<br>AYRMPRHSMKRISKEEMKSFEGRCERSGEVERTFLGTKWCGAGNTSTSYSDLGTLNNIDSCCRDHDHCDSIAAGE<br>TKYGLKNKGENSLMNCDCEVAFAACLDEVPSKTYWLLSKATEKVSIPVIKKFYFDWYGNSCYNLTCSSGRSLRNN<br>ECAKPVAEYNGPTGFLNPYING *	Phospholipase A2<br>(218546750)
sdc14190_g1_i2	RLKEGDLKIKGRRPSYYFRYNNRVSLKTRVDTVLSWLDLPHDSRPSFITFYINEPDHTSHRHGPFSQQVEAALRKIE<br>NLFQQLMAGLQTRGLINCVNIIIVGDHGMSDVECDKVILLKDYVNSSDVYFMAGPVGRIRPKHYAESSIQNIVNEF<br>RCHRPEVLVYRKSELPKHYHFSANRKIEPVIIDLPSKWTIAQSVDPNYCGGGAHGYDNLNPDMNTIFVAYGPSFKR<br>NLVVKPFLNVELYEMMSELIQVTPEPNNGTYGSLHNILRNPQLLPNTPTPKAFTTCVISGLQRRNGNLSGCNCKEN<br>FSTGGTYLPNERRRNEEALIPWGAPILKGTDSLAVCHLVNSDYVTAFHKELKLPLWTSFTISKKNSLFSGTSKFTSSK<br>CWLADGRIPAENLANCTHYRELAKEYPGLQQRPLFPIAFASSETSELSVQLMTNAVPMYQHFRSEMWLQFLILLSK<br>WSHKFSSLNVVMGPAFDINGNGVRPHIKDLMKDDKIPVPTHYFAVVTYCHSTDIPIETCRSADIEVLSFLIPHRNYP<br>DNCQNVNEYFLKHSAKVKDVEIITGLNFFTQLSPYTAISLRTHLVEELWNV *	Venom phosphodiesterase 2<br>(387014162)
	HYALURONIDASES	
sdc14512_g1_i1	MSSIFFVCVILNWVFSTSDANFEVFWNVPSSLCSIKFGVNMTQTVLKHNILVNNGETFTGDKITLIYESGIGKYPHID<br>PKKGDVNGGLPRLDSLDEHLKLAEKDIQKIIPNPNFNGLGIIDWEAWRPIWEYHWGSLGVYKNKTLDLVKKEHPS<br>WSKQIIESTAKNLWESTAKQWMLKTLELAKNLRPHGMWCYYLFPDCYNYFGKDQPSQFFCNNMIKSNNDRLSW<br>MWDASAALCPSIYFIEFQQKYNESQRIWYLYGKLSEAVRVSRPHTQIYPYINYMVHVSRIPVPKDQFWKMLSMTAS<br>LGLDGAVIWGSSGYLQSKKTCEDLEAYVENIIGPAVTTISSNVKRCSQTVCNGRGKCTWPKEPFTSWKYLIDSKGH<br>DFDAQNIICRCQNHNGRYCS *	Hyaluronidase 1<br>(597439573)
sdc14647_g1_i1	MLLILIISMFSLSALGVDFNVYWHVPSSPCSERFNINVTHKLLKCNVLVNSGEKFRGDKIVWFYEYTFGRFPYMDDV<br>NSYMDGGIPQLGNLTSHLEWAERHVEEIIPNPNFDGIAVIDWREWRPIYDYNWEVYQNVTKELVRKNNPSIREEEI<br>ESTARIQWEEAAKKWLLETLKLVKRMRPKAKWCYYSFPDCYNHQRGDVPHDFTCRKEIQRHNDRLSWLWKEST<br>ALCPSIKLGSREEKYDDSERQWYLYGRLSEARRVASPNTLIYPFVSSFRNPNWESLDSFLDMIGAPASMGFDGAVIS	Hyaluronidase 1<br>(597439573)

	GSSKYIRDKNDCEELDWYVTAVICPAVTKVYTQFHKCSRTVCTNDGRCTWPYEIFNSMDYMLYDSCRNFNSKAVF CRCEENEGRYCDKYYYNDYY *	
sdc14647_g1_i5	MLLILIISMFSLSALGVDFNVYWHVPSSPCSERFNINVTHKLLKCNVLVNSGEKFRGDKIVWFYGLKFGYMKTDNG VDYYVNGGIPQFANLTYQLKWVETNIAELIPNPNFDGIAVIDWREWRPIYDYNWEVYQNVTKELVRKNNPSIREE EIESTARIQWEEAAKKWLLETLKLVKRMRPKAKWCYYSFPDCYNHQRGDVPHDFTCRKEIQRHNDRLSWLWKES TALCPSIKLGSREEKYDDSERQWYLYGRLSEARRVASPNTLIYPFVSSFRNPNWESLDSFLDMIGAPASMGFDGAVIS GFSKYIRYKNDC	Hyaluronidase 1 (597439573)
sdc14647_g1_i2	MLLILIISMFSLSALGVDFNVYWHVPSSPCSERFNINVTHKLLKCNVLVNSGEKFRGDKIVWFYEYTFGRFPYMDDV NSYMDGGIPQLGNLTSHLEWAERHVEEIIPNPNFDGIAVIDWREWRPIYDYNWEVYQNVTKELVRKNNPSIREEEI ESTARIQWEEAAKKWLLETLKLVKRMRPKAKWCYYSFPDCYNHQRGDVPHDFTCRKEIQRHNDRIPSWI *	Hyaluronidase 2 (573016564)
sdc14647_g1_i3	PCSEMFNINVTHKLLKCNVLVNNGEEFWGDKFVWFYELKFGMFPYIHTVNGEHIYNDGGIPQFDNLTFHLAWAE TEIEQLTDPNFDGIGVIDWRQWNPIYDYNLGSKSIYKKLTKELVKENNPSIREEEIESTARIQWEEAAKKWLLETLKL VKRMRPKAKWCYYSFPDCYNHQRGDVPHDFACRKEIQQHNDRIPSWI *	Hyaluronidase 2 (573016564)
sdc14647_g1_i4	PCSEMFNINVTHKLLKCNVLVNNGEEFWGDKFVWFYELKFGMFPYIHTVNGEHIYNDGGIPQFDNLTFHLAWAE TEIEQLTDPNFDGIGVIDWRQWNPIYDYNLGSKSIYKKLTKELVKENNPSIREEEIESTARIQWEEAAKKWLLETLKL VKRMRPKAKWCYYSFPDCYNHQRGDVPHDFACRKEIQQHNDRLSWLWKESTALCPSIKLGSREEKYDDSERQW YLYGRLSEARRVA	Hyaluronidase 2 (573016564)
	**La 1 LIKE PEPTIDES**	
sdc12897_g1_i1	MRSVIMEPPYTAVLLGLLLAFSVFNFSMGFGESCKGGPYSIIPVGQEMTDPTTCTSYKCINYNRKYVLQTSTCATVK PPCKGMGSFQGNRFPNCCPIVTCTGG *	La1 like protein 13 (430802824)
sdc14036_g1_i1	MKYLYFAVFFGCLCSLISVSMGGGEVCVLGGMTIPVGQEKKDPKSCVLYKCVGVNNRVLIEKSVCQPQVKGRGCK SVDGPPSAPFPDCCPTSLCRGKQWDR *	La1 like protein 15 (430802826)
sdc10164_g1_i1	MLFSDRTLRCILIISCLFFTISDAYMYYQPQEDGSVCIDGSGKERQLNDVWYDDSKCEENACLKGRGTPYIQTYGCG VVEPG	Putative secreted protein (240247657)
sdc13004_g1_i1	MTLLNLVLICMYFIVTNAYMYYDPQELGSVDCTDRRNGVHHPVGEQWYIDELCESNTCKQFKDLSLAIITSGCGV VEPGPGCKLVRGTGSYPDCCLDEVC *	Putative secreted protein (240247657)
sdc7328_g1_i1	DIPIYEEKQDPAKCTLYKCEKDAGRIVLNTVTCAPQEPKTGCRNVDSPVELPFPDCCPLVVCNAPVYGG *	SV_SVC-Cer 1 (522802545)
sdc14589_g1_i1	MKKLIFSLVLICIFFIAIEAYTYIAPQEPGSVDCTDELGVHHPLGEVWYNEERCERLVCQGYGNGLSITGSGCGIVSA PGCKLVKGSGSYPKCCPKPVCRKR *	Toxin like protein 14 (430802832)
sdc5116_g1_i1	MDRNIILFFVLPFVVLGNDDLRFITYKNDVVFPLTEGKCNAGSGRLINQGDTWYSDEYCEKFKCLRTGILGHVEVR GCAPVYPIRPNCTVVHHKGVYPDCCDGDIVCNEQQEPKSDVEMAELIRALLEESNKK *	Venom protein 7 (149134066)
	**VENOM COMPONENTS**	
	VENOM PROTEINS	

sdc1837_g1_i1	MKLVLVLACLIAFHAVAGQESCSKSSDCEDGECCLEDFYQDTAKCEKKSESRKFCSEESAQSADDRYLFYCPCKDG LSCIVTEN	BLTX651 (671759197)
sdc11506_g1_i1	LLIILSVVLVVVYARDKSECELHREREQKRIGVGKLVPECEENGDYKGLQCHGGTRFCQCWDIKGHPITPPSMFLKS CECHREKKIAEEGDIIGAFIPRCKKNGKYEKKQCWASTGTCWCVDEDGKKTSDPTRDDIDC *	Ctenitoxinlike-lyc-1 (522802644)
sdc13673_g1_i1	MKTFISIVVLISIAGTLAQKKTECQESREKALKSNARIKAIIPVCDTNGDYAGLQCHEGSKFCSCWRKDGTPITQPSG KIKACECHRQKDESSKKGLIGAFIPQCAEDGKFHKKQCWGSTGHCWCADPETGKNTTQRVRGKLSC *	Ctenitoxinlike-lyc-1 (522802644)
sdc11734_g1_i2	MKTVIAIVLACFVAVVVSEETACQRARERQLQNRNSVIVNRCDENGNYEALQVYSDGSWKICFTPDGDVIQGPSR TIEYCECFKTRYELSKQNLPSEKLPDCKATGEFVALQSDGVSHWCVDKVTGEKTTEPRPLSRSITCD *	Thyropin 2 (629510299)
sdc14254_g1_i1	AFEMWYGVWLVGLVGLAAGAKPDCPPCKKESCQAPLNCLAGLVKDICDCCFVCGRTEGERCDNLALPLPYRRK YGYCGENLECRLRTDLAPEDPDEGICVCLKQEAICANDGNTYENECKLTEARYKLRDLNYLRAVSRGPCRSAPKII SPPETTKNRTGGSAALSCEVTGWPIPVIEWKVDHGDGIMSPMPSDRPRISVQTRGGPGNYEVTSWLQMQNLELRD HATYWCIARNDEGESTASAQIRIVNFPRFNEI *	Insulin like growth factor binding related protein 1 (351720390)
sdc2109_g1_i1	SVTKMVIYVLIILMTTAGLCRGAAIVDGELICSCDDVLCQQIGNCPLGEVKGICGCCNECAHDVGEPCGSLYNYGG ICGVGLKCEPNEFKQLPG	Orphan peptide AbOp5 (723219378)
sdc14152_g1_i1	DICRRSQKTQIHIFALSSYRVNRIANRCTMIRLVIFSVLVVSIYSLSCPCWREEDPTKYCGPPPENCAKGVTTDVCGC CPVCYKVEGEVCGGPWGVYGDCGEGLVCDKGASLEEGEFDQREGVCKPE *	Tbah01400 (757181026)
sdc14453_g2_i1	MLFRIAVYSLLLVVCVYSLSCPLCWENPSVCGTTPTDCKLGLTKDACGCCDVCYKIEGEKCAGPFRTSGECGKGLK CVISDDEPQHNRFQAEGTCMPEN *	Tbah01400 (757181026)
sdc14453_g2_i2	MLFRIAVYSLLLVVCVYSLSCPLCWENPSVCGTTPTDCKLGLTKDACGCCDVCYKIEGEKCGGPWRTSGNCGKGL KCEIPKDLPKHIQRQAVGTCKPE *	Tbah01400 (757181026)
sdc14388_g1_i1	MNRYWLISFILLGILMGGMAQDCMQCGTYQCYTTPAECPAGTVTDMCDCCLVCAKDEDEECGGLWDMRGKCG EGLTCVKENGDDENSVGVCKKE *	Tbah02469 (757181036)
sdc11340_g1_i1	SRESKVTNKMDLRLFVLTVLIVSCHCLPSKRNDACGPFPSNCRVGVTKDIDGCPVCTKDVGEECGGSWNAYGICN VDLICQTNGQSDNDYELPIGICVPARRFSSRNILKRMIGGRH *	Venom insulin like growth factor binding protein 1 (149134032)
sdc14478_g1_i1	MNKFWMISFILLGIFTSSMALSCMPCGEYECEPLPAHCPAGIGRDICGCCPVCAQAENEICGGPWDMNGRCGDGL TCVKEKHDDEDYLEFNQSGVCKKV *	Venom insulin like growth factor binding protein 1 (149134032)
sdc14478_g2_i1	VSLETKHLKNINMNKFWTISFVLLGIFTSSMALSCLPCYERECEPLPAHCPAGIGKDICYCCPVCAQAENEECGGP WDMLGRCGDGLTCVKEEDDYIDEFNRGGICKKV *	Venom insulin like growth factor binding protein 1 (149134032)
sdc14478_g1_i2	MNKFWMISFILLGIFTSSMALSCTSCGEYECLPLPAHCPAGIGRDICGCCPVCAQAENEICGGPWDMDGRCGDGL TCVHEKNDDDDYIEVCKKV *	Venom insulin like growth factor binding protein 1 (149134032)

sdc30596_g1_i1	CPNTSSFDKMKSLLITAFFALACTTVHTFSNRELEELFCSLPNHLAARWIDCILEDAAESISKSANVVHTCVDEFWD VKGLGDSLYSMQCNWDIRRDDNVGECIMEKAKSLDFDQPPTEEEFLAVKNRIEPCLFTAK *	Venom protein 29 (317412178)
sdc25935_g1_i1	QIFSENMGKLLFICLILLGISISAMALSCPRCEPDKCSPPPADCPAGIVKDVCHCCFRCAQAENEKCGGPWGIAGKC GDGLTCVKETREGESPLGSIHRIGVCQKE *	Venom protein 302 (317412179)
	CAP SUPERFAMILY	
sdc13900_g1_i1	MAPTIVPVFIIWAVTINCIDSVDLCDERYTNITKEHTMCKPVNEDCNFLRVGKIFSKQLLRTHNDIRNSIRKFVGKEY KLATNMELMEWDEELYAMARMHTLQCVDKPDCNLCHQIDDFPVEQNFAVKTFKRSEVKHNGPVKRFQTVIKG WAAELQSYDPDVVNNLTITDKLPTHWTNILRATTVFVGCASMNFYEDEPSVFKEVYVCNYGPANLTEGEQIYKTG NKSCSDCEDNGVCDEEFKNLCVPSELEEGSTENFPEGETTNLEEANTQETTYIEEGSTENFPSEETTYIEIMESSPSKE NISREVPETYTSIVQENETYFVTEVYTETSMEEYYTTELVPSEEKK *	CAP-Iso-1 (522802633)
sdc13157_g2_i1	INYFYKQTTKLKVMILGIIIHFIIGPLPTSNTFSTEKLFIFSKGSVICEIGKNCNSLLTSLNGNIIHDVNVERQRLTIGFYE GLPIATNMLKMIRDCDLEAKAQQFAETCALQKSSHCRPPNDAEGEMNISVRKYNMNNQGKDVYDRLRDQVLR WKPPMGKEYKGPLTQLVKSYKLEDINNREWANYVRANVWKVGCGIADVEMPNATFMEVFVCYFGSPKIRDGDE LYLAAGCVQCSQCPNGTRCRNGLCELEEFFGWKNILKCQEKPYNFAPSTNYERSPENGPCYEVATTTAEITPRPTT PALLTMEEEIITEDILKFSLIEDSAQACVDNTCSPGWSYNSGRVWSSFQIAR *	CAP-Lyc-1 (522802698)
sdc13157_g2_i2	INYFYKQTTKLKVMILGIIIHFIIGPLPTSNTFSTEKLFIFSKGSVICEIGKNCNSLLTSLNGNIIHDVNVERQRLTIGFYEG LPIATNMLKMIRDCDLEAKAQQFAETCALQKSSHCRPPNDAEGEMNISVRKYNMNNQGKDVYDRLRDQVLRWKP PMGKEYKGPLTQLVKSYKLEDINNREWANYVRANVWKVGCGIADVEMPNATFMEVFVCYFGSPKIRDGDELYLA AGCVQCSQCPNGTRCRNGLCELEEFFGWKNILKCQEKPYNFAPSTNYERSPENGPCYEVATTTAEITPRPTTPALLT MEEEIITEDILKFSLIEDSAQACVDNTCSPGWSYNSGKAEIKGEGICMATVAFREKINLSGYSCLKIQYSLTSGDEMASE VVAFAFEVNNKMGTIQKRIKYENGNNHMFLDIPWPGVEFRVGFGARSKLHDEQFEVIVDEVIILRGNCPQI *	CAP-Lyc-1 (522802698)
sdc13879_g1_i1	MKQNHSMFFAMKAMVLIVIRFLIFDVFVSKGVIAGSARLIFKDSNYKDCHIGTSECNFADIHKWFLKNHNSIRSTG TAGQSATNMLMMEWDQKLTDAAQSYAEECLPVSCLPERANFSGEVNTDIREHPYQSAASLSERIRKVLKYWKEE VDDYDSDMLTNQIRSYKIVSRDMQRWANVVRATTWKVGCGIVDIYSGKENPFNEIIVCFYKNAKLKEGEELYKIG KPCTECPQGTSCSEYLKTLCEVNPGKCPITSNERTIEESCSTKGNTVWRCSVEQGTEDCLAERACSSFWEIDVIGKFK KISVSGMCSSVNIFHKEIQIDEPACFIFEYIKEPTLSQAVKSEVTGFILQSNGQYSEKVTASDDAKEWTPIKVDIPWTGI AVQVGVGVRSRSPPTAGKQDIKVRNFSVVTGKCT *	CAP-Lyc-1 (522802698)
sdc3852_g1_i1	VTKQAAIMIPVLLTSLTLTCHVVLSQSCPEIYLRFSKDHTYCRRSTCKVMKSGVTEEDKKIILDMHNKYRNKIALGQ ETEPQQQPPAANMMQMEWDDELAKVAQAHASLCKFDHDTGPQRQVENFNVGQNLYITMMSKKINWKKASV WYTSEVKLFYPQYREPYVFGAWGHFSQMIWAKTWKVGCGMAMYYEENMDKILYTCNYGPAGNMQGGTTYIKG NPCSQCPKNTQCSNEYRGLCKSLTPDGPQEDNSKSSNDLILYCDFSTNDPAECKNVQITGSKQFQTRKIYSGEYKTV VLNGGDSITIKLGKAQSEKGICPFIYGYFGPNRDGDAKQSAVSIGFSAPGINFGQPTKIEYGGSSFWTIGMQMQFNQ EMESTIKLEAYPGAAPQYFNVKAFGIGRGKCPKF *	CAP-Uro-1 (522802590)
sdc11413_g1_i1	NMMQMEWDDELARVAQAHANLCVFKHDKGKERAVENFPVGQNLLKFSPAIRKWEAINSWYKQEVCFFLPEYI KPFHSGDYGHFSQFTWATTWKIGCGFTAYSEGGKNFSLYTCNYGPTGNILQGTQYIVGEPCSQCPENTECSKPYPG	CAP-Uro-1 (522802590)

	LCKSKTRDGPQMTKPPSEDYALYCDFSSDDPDKCRDVKISGSRNFTTRHIYSGNYKTVILEGGEYVTIDLGMAKNE<br>DGICPFVYIRCGPNNATETIGLVMEISFSVPGNTPQSPMKIYPNAGTSFSPIGVHMRSNFEFKSTITLRAKDDGAPQF<br>VDVRIWGIRKSDCKNPPL *	
sdc14327_g1_i1	GIFLFHLCSEMERVLCTVLFLQILWWMSAGCKYERVGRHHTMCIYSAHACPNSQLIRSGGITTRDKNLIVKIHNQVR<br>SKVALGKVHGLPPAADMRVMTWDNELAKIAQRWADQCTEGHDKLRDTERESVGQNVALRWSYDHKDPLLKDK<br>PDWPFSIDLWSKEYDEFGFSSSHISPFVFKYSVGHYTQMIWAETHKIGCGFTYYKHPQKGYTKIYVCNYSPGGNIIQGT<br>MYKTSPRGATCSDSSLQLSREYKGLCEKSRRSRIR**R**RNSNRRK**R**VISQTRHERSRTFQFSKQQKSRNARRKGSTN *	Putative scp tpx 1 ag5 pr1<br>(757180984)
sdc11413_g2_i1	MKVLPIVSCLLLTQCSSILCLSCSETDYGKYDEDTRDIKIPISLWRTPTSGRNKVETLESKECPLLYQRYSSNHTYCKTS<br>TCDIIFKGVNSGEKKMILHIHNSLRRKLANGQETRYRQLPSAANMMQMEW	Putative cysteine rich secretory peptide<br>(757180946)
sdc11413_g2_i2	YMVFEINKSDFSSYSTRMKVLPIVSCLLLTQCSSILCLSCSETDYGKYDEDTRDIKIPISLWRTPTSGRNKVETLESKEC<br>PLLYQRYSSNHTYCKTSTCDIIFKGVNSGEKKMILHIHNSLRRKLANGQETRYRQLPSAANMMQMEW	Putative cysteine rich secretory peptide<br>(757180946)
sdc14601_g1_i1	MNTKGCIIGYVPQTLMFVLIATVVSSLGKNICDTRYRNIHPEHSMCKTRNYTCKFRSGIEENVQPLLDLHNHIRNSI<br>QKVVGNNYFEGRNMMNMQWDRELYLIAQRHVLQCTDLPDCSQCHQIDRFHVEQNFAVNTFSSVNHSFNGSVE<br>RFK**R**VIRDWASELRKYNPCVVERFQFLGLPTNWTNIFRATTLKVGCASVTYDSQTKGRFTEIYVCNYGPALLTEGV<br>EIYKPNYQNCNECSNNIKCNTSTPLQSRDLRQQLGDYQQRARKSQRQLIARAPVIGKSKRQIITPTTTTTTEQFQNH<br>PTEIDLLVDSGIIYPSTPETEAHTTETTKITITATPVPISEIITTEKKIPTKDTATPTVKSSTETEIDLLIASETIFPSISTTESST<br>IGTAKPIPTTTPVSTSRSANPEYNIYTQITNTETIIPSPDTEVDLIEASEIISTSSTTEEASMTGVTKPDIGLVEVSEIISTSST<br>TESSISEATILD	Tbah00853<br>(324497915)
sdc14601_g1_i2	MNTKGCIIGYVPQTLMFVLIATVVSSLGKNICDTRYRNIHPEHSMCKTRNYTCKFRSGIEENVQPLLDLHNHIRNSI<br>QKVVGNNYFEGRNMMNMQWDRELYLIAQRHVLQCTDLPDCSQCHQIDRFHVEQNFAVNTFSSVNHSFNGSVE<br>RFKRVIRDWASELRKYNPCVVERFQFLGLPTNWTNIFRATTLKVGCASVTYDSQTKGRFTEIYVCNYGPALLTEGV<br>EIYKPNYQNCNECSNNIKCNTSTPLQSRDLRQQLGDYQQRARKSQRQLIARAPVIEEYMEVDIPEQTRSGLLFLNTI<br>YIEEPSCFIFSYKKNAVPIWSPLTSSVYGIAVRTESNDYVMVQRDTDVYDWMHVFLDIPWIKVFIQVGVGIRTDYGA<br>GEQHIQIKDLVLRRGTCSAL *	Tbah00853<br>(556052306)
sdc11381_g1_i3	MESMIIIVTTCLIFDIFVSNGVTDDSAYLIFKHNTYKDCIIGENGCNLKEVQMMFLERHNFMRSRIAGSDSTTEPDAA<br>NMLMMEWDQSLANDAQNYAQNCLERGCQPEEAGFNGEVNIYTNSYDKADAHVESLPTRISNTIWSWVYDSVH<br>GHSSEDIKSLLHSYKSDSPTMHQWANVIRATTWKMGCGIADILISPGANQFSEIIVCFYKNSKLNDGDELYKIGQPC<br>TNCPPGTICSYKESLSNLCESIPGYCPITSNELTVEDSCYRSELQRTSIWNCSAEDDTENCRPERPCASVWKIDPYGKF<br>KKITVSGMCTSANMYQKEIEINEPACFIFEYIKEPSVNQAIKSTVTGFVLQSNGKYSEKVTVSEDVDKWTPVKLDIY<br>WTGIPLQIGISVRSRSTPTTGEQEIKIRNFLIVTGSCA *	Venom allergen 5<br>(675383069)
sdc11381_g1_i2	MESMIIIVTTCLIFDIFVSNGVTDDSAYLIFKHNTYKDCIIGENGCNLKEVQMMFLERHNFMRSRIAGSDSTTEPDAA<br>NMLMMVIRATTWKMGCGIADILISPGANQFSEIIVCFYKNSKLNDGDELYKIGQPCTNCPPGTICSYKESLSNLCESI<br>PGYCPITSNELTVEDSCYRSELQRTSIWNCSAEDDTENCRPERPCASVWKIDPYGKFKKITVSGMCTSANMYQKEIE<br>INEPACFIFEYIKEPSVNQAIKSTVTGFVLQSNGKYSEKVTVSEDVDKWTPVKLDIYWTGIPLQIGISVRSRSTPTTGE<br>QEIKIRNFLIVTGSCA *	Venom allergen 5<br>(675383069)

Table S2. Amino acid sequences of peptides.

	Transcript	Sequence (MS/MS)
Enzymes	sdc14619_g1_i3	GYDWTDALAYAK
		EFADAFFEFR
		SLGGWPLLGETIK
		IGEIIEGSYLENTITIAK
		TETIANYIGWTIVNGYGR
		ANYYFGLPLGNLFINNR
		LHLELNTEQIDILLLLNK
		KLnGFYEKIGEIIEGSYLEnTITIAK
		TcKLLEQnTTKTETIAnYIGWTIVnGYGR
	sdc14619_g1_i1	LGEIIEGSHLENVINIVK
		HFYFVTIK
	sdc14393_g1_i1	NDEHITLLHcDcEEAFDK
		FSLTFIK
	sdc14212_g1_i1	ELYVNFEPLPGQK
		AFDScLQNAANK
		YGLTNDGEYTLLNcncDK
		FAYFTVYAPK
CAP	sdc3852_g1_i1	LEAYPGAAPQYFNVK
		QSAVSIGFSAPGINFGQPTK
		EPYVFGAWGHFSQmIWAK
	sdc13900_g1_i1	mHTLQcVDKPDcNLcHQIDDFPVEQNFAVK
		LATNmELmEWDEELYAmAR
La1-like	sdc13004_g1_i1	YmYYDPQELGSVDcTDR
		RnGVHHPVGEQWYIDELcESNTcK
		DLSLAIITSGcGVVEPGPGcK
		NGVHHPVGEqWYIDELcESnTcK
		GTGSYPDccLDEVc
	sdc14036_g1_i1	SVDGPPSAPFPDccPTSLcR
		GGEVcVLGGMTIPVGQEK
	sdc12897_g1_i1	GGPYSIIPVGQEMTDPTTcTSYKcINYNRK
		KYVLQTSTcATVKPPcK
	sdc14589_g1_i1	YTYIAPQEPGSVDcTDELGVHHPLGEVWYNEER
NaTx	sdc14462_g1_i1	HGAHGGScYSIPSR
		KQEAGYGYcYDR
		YcWcEGK
		KGGSTIPcLK
		HGGDSYcVDVcAK
		GDYVWcGLPGGENKEcEDVcR
		GGSTIPcLK
	sdc14462_g1_i2	IcAEHGAHGGScHGLPSR
	sdc14462_g2_i2	DKnGDWIWcGLPGGK
		YcWcEAP
	sdc15193_g1_i1	TcYcEGLTITK
KTx	sdc14273_g1_i2	SGEEPPIWPcR
	sdc13949_g1_i1	FGKGGKcDGPK
		mRAPTGGcPFSDALcANYcKK
Sc	sdc14222_g4_i2	GcQETVQQIGYcHGTK
		LDSScILGHDR
		AIDEFVPmIPVPPSVSGALQK
	sdc14222_g4_i1	LDTPcIFGIDKK
		AIDVLTPmIPVPVVGGIVNK
		cKcGKPLnYK

NDBP	sdc12606_g1_i1	IGTNPPQAAT<br>IWNSDFVK
	sdc4010_g1_i1	NIWNSDIVK
	sdc14358_g12_i1	GFWGDVWSGIK
	sdc14358_g5_i1	IFGAIWNGIK
	sdc6540_g1_i1	GFWGNVWEGIK
	sdc28695_g1_i1	VLPSLLSSGK<br>FWGFLAK
	sdc13544_g1_i1	YFWTYTFPK
	sdc14106_g1_i1	FPFFLASLIPSAINLVK

Table S3. Twenty two sequences of Calcins and putative Calcins isolated from the venom or deduced from cDNA or transcriptome analysis of 14 scorpion species in 12 genera and 8 families. Code = UniProt accession numbers; except for those with the names given in the original publications (listed in the references). * Possible not true Scorpion calcins.

Code	Species	Family	Reference
Q8I6X9	*Mesobuthus martensii*	Buthidae	UniProt
B8QG00	*Hoffmannihadrurus gertschi*	Caraboctonidae	UniProt
Ctyc5	*Chaerilus tryznai*	Chaerilidae	1
Ctric74	*Chaerilus triscostatus*	Chaerilidae	1
Ctric58	*Chaerilus triscostatus*	Chaerilidae	1
Ctryc59 *	*Chaerilus tryznai*	Chaerilidae	1
Ctric33 *	*Chaerilus triscostatus*	Chaerilidae	1
Ctryc40 *	*Chaerilus tryznai*	Chaerilidae	1
Ctric36 *	*Chaerilus triscostatus*	Chaerilidae	1
P60252	*Opistophthalmus carinatus*	Scorpionidae	UniProt
P60253	*Opistophthalmus carinatus*	Scorpionidae	UniProt
FD664155	*Heterometrus petersii*	Scorpionidae	UniProt
P59868	*Pandinus imperator*	Scorpionidae	UniProt
P60254	*Scorpio palmatus*	Scorpionidae	UniProt
Smp33	*Scorpio palmatus*	Scorpionidae	2
FD664670	*Scorpiops margerisonae*	Scorpiopidae	UniProt
GH547959	*Scorpiops jendeki*	Scorpiopidae	UniProt
sd9999	*Superstitionia donensis*	Superstitioniidae	This study
sd13987	*Superstitionia donensis*	Superstitioniidae	This study
L0GBR1	*Urodacus yaschenkoi*	Urodacidae	3
ViCaTx1	*Thorellius intrepidus*	Vaejovidae	3
VpCaTx1	*Mesomexovis punctatus*	Vaejovidae	3

Table S4. Ninety six sequences of Scorpines and putative Scorpines isolated from the venom or deduced from cDNA or transcriptome analysis of 34 scorpion species in 22 genera and 10 families; one sequence of the Pfam domain "Toxin 38" from a mite (Ixodae); and one sequence of a Potassium channel α toxin from one scorpion species. Code = UniProt accession numbers, except for those with the names given in the original publications (listed in the references).

Code	Species	Family	Pfam	Reference
Q6XLL8_KTx6	*Opisthacanthus cayaporum*	Hormuridae	Toxin 2	UniProt
A0A023FQN5_acari	*Amblyomma cajennense*	Ixodae	Toxin 38	UniProt
P69939	*Androctonus australis*	Buthidae	Toxin 38	UniProt
A0A0K0LBZ4	*Androctonus bicolor*	Buthidae	Toxin 38	UniProt
A0A0K0LC02	*Androctonus bicolor*	Buthidae	Toxin 38	UniProt
A0A0K0LC05	*Androctonus bicolor*	Buthidae	Toxin 38	UniProt
A0A0K0LC06	*Androctonus bicolor*	Buthidae	Toxin 38	UniProt
A0A0K0LC08	*Androctonus bicolor*	Buthidae	Toxin 38	UniProt
A0A0K0LC09	*Androctonus bicolor*	Buthidae	Toxin 38	UniProt
A0A0K0LC11	*Androctonus bicolor*	Buthidae	Toxin 38	UniProt
A0A0K0LC14	*Androctonus bicolor*	Buthidae	Toxin 38	UniProt
A0A0K0LC17	*Androctonus bicolor*	Buthidae	Toxin 38	UniProt
A0A0K0LCI9	*Androctonus bicolor*	Buthidae	Toxin 38	UniProt
A0A0K0LCJ0	*Androctonus bicolor*	Buthidae	Toxin 38	UniProt
T1E6W5	*Australobuthus xerolimniorum*	Buthidae	Toxin 38	UniProt
B8XH40	*Buthus occitanus*	Buthidae	Toxin 38	UniProt
B8XH36	*Buthus occitanus*	Buthidae	Toxin 38	UniProt
T1DEK6	*Isometroides vescus*	Buthidae	Toxin 38	UniProt
T1E6Y1	*Isometroides vescus*	Buthidae	Toxin 38	UniProt
T1E6Z4	*Lychas buchari*	Buthidae	Toxin 38	UniProt
C6ZH27	*Lychas mucronatus*	Buthidae	Toxin 38	UniProt
D9U2A7	*Lychas mucronatus*	Buthidae	Toxin 38	UniProt
D9U2B1	*Lychas mucronatus*	Buthidae	Toxin 38	UniProt
P0CI42	*Lychas mucronatus*	Buthidae	Toxin 38	UniProt
P0CI49	*Lychas mucronatus*	Buthidae	Toxin 38	UniProt
P0CJ45	*Lychas mucronatus*	Buthidae	Toxin 38	UniProt
A9XE59	*Mesobuthus eupeus*	Buthidae	Toxin 38	UniProt
A9XE60	*Mesobuthus eupeus*	Buthidae	Toxin 38	UniProt
P0CH57	*Mesobuthus eupeus*	Buthidae	Toxin 38	UniProt
A0A088D9S1	*Mesobuthus eupeus*	Buthidae	Toxin 38	UniProt
A0A088D9U2	*Mesobuthus eupeus*	Buthidae	Toxin 38	UniProt
A0A088DAE8	*Mesobuthus eupeus*	Buthidae	Toxin 38	UniProt
A0A088DB26	*Mesobuthus eupeus*	Buthidae	Toxin 38	UniProt
A0A088DB53	*Mesobuthus eupeus*	Buthidae	Toxin 38	UniProt
E4VNZ9	*Mesobuthus eupeus*	Buthidae	Toxin 38	UniProt
E4VP14	*Mesobuthus eupeus*	Buthidae	Toxin 38	UniProt
E4VP34	*Mesobuthus eupeus*	Buthidae	Toxin 38	UniProt
**Code**	**Species**	**Family**	**Pfam**	**Reference**
E4VP56	*Mesobuthus eupeus*	Buthidae	Toxin 38	UniProt
E4VP57	*Mesobuthus eupeus*	Buthidae	Toxin 38	UniProt
A0A059UI30	*Mesobuthus gibbosus*	Buthidae	Toxin 38	UniProt
Q9N661	*Mesobuthus martensii*	Buthidae	Toxin 38	UniProt
Q9NJC6	*Mesobuthus martensii*	Buthidae	Toxin 38	UniProt
A0A0C9RFQ9	*Tityus bahiensis*	Buthidae	Toxin 38	UniProt
A0A0C9S3A8	*Tityus bahiensis*	Buthidae	Toxin 38	UniProt
Q0GY42	*Tityus costatus*	Buthidae	Toxin 38	UniProt
Q5G8A6	*Tityus costatus*	Buthidae	Toxin 38	UniProt
Q0GY43	*Tityus discrepans*	Buthidae	Toxin 38	UniProt
Q0GY44	*Tityus discrepans*	Buthidae	Toxin 38	UniProt
P69940	*Tityus serrulatus*	Buthidae	Toxin 38	UniProt
P86822	*Tityus serrulatus*	Buthidae	Toxin 38	UniProt
P0C2F3	*Tityus stigmurus*	Buthidae	Toxin 38	UniProt
P0C8W4	*Tityus stigmurus*	Buthidae	Toxin 38	UniProt
K9LZ65	*Tityus stigmurus*	Buthidae	Toxin 38	UniProt
Q0GY45	*Tityus trivittatus*	Buthidae	Toxin 38	UniProt
Q0GY46	*Tityus trivittatus*	Buthidae	Toxin 38	UniProt
T1DMR0	*Cercophonius squama*	Botrhiuridae	Toxin 38	UniProt
T1DP99	*Cercophonius squama*	Botrhiuridae	Toxin 38	UniProt

T1E6W8	*Cercophonius squama*	Botrhiuridae	Toxin 38	UniProt
P0C8W5	*Hoffmannihadrurus gertschi*	Caraboctonidae	Toxin 38	UniProt
Q0GY40	*Hoffmannihadrurus gertschi*	Caraboctonidae	Toxin 38	UniProt
Q0GY41	*Hoffmannihadrurus gertschi*	Caraboctonidae	Toxin 38	UniProt
Ctri27	*Chaerilus tricostatus*	Chaerilidae	Toxin 38	1
Ctri13	*Chaerilus tricostatus*	Chaerilidae	Toxin 38	1
Ctri9164	*Chaerilus tricostatus*	Chaerilidae	Toxin 38	1
Ctry44	*Chaerilus tryznai*	Chaerilidae	Toxin 38	1
Ctry51	*Chaerilus tryznai*	Chaerilidae	Toxin 38	1
Ctry22350	*Chaerilus tryznai*	Chaerilidae	Toxin 38	1
C7G3K3	*Liocheles australasiae*	Hormuridae	Toxin 38	UniProt
C5J891	*Opisthacanthus cayaporum*	Hormuridae	Toxin 38	UniProt
P0C2F4	*Heterometrus laoticus*	Scorpionidae	Toxin 38	UniProt
Q5WQZ7	*Opistophthalmus carinatus*	Scorpionidae	Toxin 38	UniProt
Q5WQZ9	*Opistophthalmus carinatus*	Scorpionidae	Toxin 38	UniProt
Q5WR01	*Opistophthalmus carinatus*	Scorpionidae	Toxin 38	UniProt
Q5WR03	*Opistophthalmus carinatus*	Scorpionidae	Toxin 38	UniProt
H2CYP8	*Pandinoides cavimanus*	Scorpionidae	Toxin 38	UniProt
H2CYQ1	*Pandinoides cavimanus*	Scorpionidae	Toxin 38	UniProt
P56972	*Pandinus imperator*	Scorpionidae	Toxin 38	UniProt
P0DL47	*Euscorpiops validus*	Scorpiopidae	Toxin 38	UniProt
sdc34997_g1_i1	*Superstitionia donensis*	Superstitioniidae	Toxin 38	This study

**Code**	**Species**	**Family**	**Pfam**	**Reference**
sdc1422_g4_i1	*Superstitionia donensis*	Superstitioniidae	Toxin 38	This study
sdc1422_g4_i2	*Superstitionia donensis*	Superstitioniidae	Toxin 38	This study
sdc20456_g1_i1	*Superstitionia donensis*	Superstitioniidae	Toxin 38	This study
sdc4553_g1_i1	*Superstitionia donensis*	Superstitioniidae	Toxin 38	This study
sdc23468_g1_i1	*Superstitionia donensis*	Superstitioniidae	Toxin 38	This study
T1DEJ8	*Urodacus manicatus*	Urodacidae	Toxin 38	UniProt
T1DMR6	*Urodacus manicatus*	Urodacidae	Toxin 38	UniProt
T1E6X2	*Urodacus manicatus*	Urodacidae	Toxin 38	UniProt
L0G8Z0	*Urodacus yaschenkoi*	Urodacidae	Toxin 38	UniProt
L0GCW2	*Urodacus yaschenkoi*	Urodacidae	Toxin 38	UniProt
VpScp1p1	*Mesomexovis punctatus*	Vaejovidae	Toxin 38	3
VsScp1p1	*Mesomexovis subcristatus*	Vaejovidae	Toxin 38	3
VsScp1p2	*Mesomexovis subcristatus*	Vaejovidae	Toxin 38	3
ViScp1p1	*Thorellius intrepidus*	Vaejovidae	Toxin 38	3
ViScp1p2	*Thorellius intrepidus*	Vaejovidae	Toxin 38	3
ViScp1p3	*Thorellius intrepidus*	Vaejovidae	Toxin 38	3
VmScp1p1	*Vaejovis mexicanus*	Vaejovidae	Toxin 38	3
VmScp1p2	*Vaejovis mexicanus*	Vaejovidae	Toxin 38	3
VmScp1p3	*Vaejovis mexicanus*	Vaejovidae	Toxin 38	3

Table S5. Thirty six sequences of La1-like and putative La1-like peptides isolated from the venom or deduced from cDNA or transcriptome analysis of 23 scorpion species in 18 genera and 9 families. Code = UniProt accession numbers, except for those with the names given in the original publications (listed in the references).

Code	Species name	Family	Reference
T1DMQ9	*Cercophonius squama*	Botrhiuridae	UniProt
T1DP97	*Cercophonius squama*	Botrhiuridae	UniProt
D2CF17	*Mesobuthus martensii*	Buthidae	UniProt
A0A0K0LCC5	*Androctonus bicolor*	Buthidae	UniProt
E4VP44	*Mesobuthus eupeus*	Buthidae	UniProt
F1CJ59	*Hottentotta judaicus*	Buthidae	UniProt
F1CJA0	*Hottentotta judaicus*	Buthidae	UniProt
FE193686	*Lychas mucronatus*	Buthidae	UniProt
FD660409	*Isometrus maculatus*	Buthidae	UniProt
Ctry	*Chaerilus tryznai*	Chaerilidae	1
Ctri	*Chaerilus triscostatus*	Chaerilidae	1
C5J895	*Opisthacanthus cayaporum*	Hormuridae	UniProt
P0C5F3	*Liocheles australasiae*	Hormuridae	UniProt
C5J8B8	*Opisthacanthus cayaporum*	Hormuridae	UniProt
H2CYP1	*Pandinoides cavimanus*	Scorpionidae	UniProt
K7WMX6	*Heterometrus spinifer*	Scorpionidae	UniProt
JN315721	*Pandinoides cavimanus*	Scorpionidae	UniProt
KC140563	*Heterometrus spinifer*	Scorpionidae	UniProt
Smp73	*Scorpio palmatus*	Scorpionidae	2
FD664111	*Heterometrus petersii*	Scorpionidae	UniProt
FD664431	*Scorpiops margerisonae*	Scorpiopidae	UniProt
GH548227	*Scorpiops jedenki*	Scorpiopidae	UniProt
GH547861	*Scorpiops jedenki*	Scorpiopidae	UniProt
GH547772	*Scorpiops jedenki*	Scorpiopidae	UniProt
GH547797	*Scorpiops jedenki*	Scorpiopidae	UniProt
sdc5116	*Superstitionia donensis*	Superstitioniidae	This study
sdc7328	*Superstitionia donensis*	Superstitioniidae	This study
sdc12897	*Superstitionia donensis*	Superstitioniidae	This study
sdc14036	*Superstitionia donensis*	Superstitioniidae	This study
L0GB04	*Urodacus yaschenkoi*	Urodacidae	UniProt
L0GCJ1	*Urodacus yaschenkoi*	Urodacidae	UniProt
L0GCW8	*Urodacus yaschenkoi*	Urodacidae	UniProt
ViLa1lp1	*Thorellius intrepidus*	Vaejovidae	3
VmLa1lp1	*Vaejovis mexicanus*	Vaejovidae	3
VpLa1lp1	*Mesomexovis punctatus*	Vaejovidae	3
VsLa1lp1	*Mesomexovis subcristatus*	Vaejovidae	3

Table S6. Twenty sequences of Potassium channel κ toxins from 8 scorpion species in 4 genera and 3 families, plus 12 sequences of Potassium channel α toxins and Chlorotoxins from nine scorpion species as outgroups; isolated from the venom or deduced from cDNA or transcriptome analysis of a total of 17 scorpion species. Code = UniProt accession numbers, except for those with the names given in the original publications (listed in the references). Type = Name of the corresponding type of toxin.

Code	Species	Family	Type	Reference
P86436	*Androctonus australis*	Buthidae	Chlorotoxin	UniProt
P59887	*Hottentotta sindicus*	Buthidae	Chlorotoxin	UniProt
P83400	*Hottentotta tamulus*	Buthidae	Chlorotoxin	UniProt
P45639	*Leiurus quinquestriatus*	Buthidae	Chlorotoxin	UniProt
P0CI86	*Lychas mucronatus*	Buthidae	unknown	UniProt
A9QLM3	*Lychas mucronatus*	Buthidae	Unknown	UniProt
Q6WGI9	*Parabuthus granulatus*	Buthidae	Scorpion acidic αKTx toxin	UniProt
P86271	*Tityus serrulatus*	Buthidae	Unknown	UniProt
P0C183	*Tityus trivittatus*	Buthidae	unknown	UniProt
B3A0L5	*Tityus trivittatus*	Buthidae	κKTx	UniProt
P86110	*Opisthacanthus cayaporum*	Hormuridae	κKTx	UniProt
C5J893	*Opisthacanthus cayaporum*	Hormuridae	κKTx	UniProt
P0C1Z3	*Opisthacanthus madagascariensis*	Hormuridae	κKTx	UniProt
P0C1Z4	*Opisthacanthus madagascariensis*	Hormuridae	κKTx	UniProt
P82851	*Heterometrus fulvipes*	Scorpionidae	κKTx	UniProt
P82850	*Heterometrus fulvipes*	Scorpionidae	κKTx	UniProt
P0DJ41	*Heterometrus laoticus*	Scorpionidae	κKTx	UniProt
P0DJ36	*Heterometrus petersii*	Scorpionidae	κKTx	UniProt
P0DJ38	*Heterometrus petersii*	Scorpionidae	κKTx	UniProt
P0DJ35	*Heterometrus petersii*	Scorpionidae	κKTx	UniProt
P0DJ39	*Heterometrus petersii*	Scorpionidae	κKTx	UniProt
P0DJ34	*Heterometrus petersii*	Scorpionidae	κKTx	UniProt
P0DJ37	*Heterometrus petersii*	Scorpionidae	κKTx	UniProt
P0DJ40	*Heterometrus petersii*	Scorpionidae	κKTx	UniProt
P0DJ33	*Heterometrus petersii*	Scorpionidae	κKTx	UniProt
P0DJ33	*Heterometrus petersii*	Scorpionidae	κKTx	UniProt
P83655	*Heterometrus spinifer*	Scorpionidae	κKTx	UniProt
P0DL35	*Scorpiops jendeki*	Scorpiopidae	unknown	UniProt
sdc14251_g2_i1	*Superstitionia donensis*	Superstitioniidae	κKTx	This study
sdc13949_g1_i1	*Superstitionia donensis*	Superstitioniidae	αKTx	This study
sdc26193_g1_i1	*Superstitionia donensis*	Superstitioniidae	Alpha	This study
VmKTx2	*Vaejovis mexicanus*	Vaejovidae	κKTx	3

References

1. He, Y.; Zhao, R.; Di, Z.; Li, Z.; Xu, X.; Hong, W.; Wu, Y.; Zhao, H.; Li, W.; Cao, Z. Molecular diversity of Chaerilidae venom peptides reveals the dynamic evolution of scorpion venom components from Buthidae to non-Buthidae. *J. Proteom.* **2013**, *89*, 1–14.
2. Abdel-Rahman, M.A.; Quintero-Hernández, V.; Possani, L.D. Venom proteomic and venomous glands transcriptomic analysis of the Egyptian scorpion *Scorpio maurus palmatus* (Arachnida: Scorpiones). *Toxicon* **2013**, *74*, 193–207.
3. Quintero-Hernández, V.; Ramírez-Carreto, S.; Romero-Gutiérrez, M.T.; Valdez-Velázquez, L.L.; Becerril, B.; Possani, L.D.; Ortiz, E. Transcriptome analysis of scorpion species belonging to the *Vaejovis* genus. *PLoS ONE* **2015**, *10*, e0117188.
